# Targeting pancreatic cancer with natural compounds: molecular mechanisms, preclinical evidence, clinical perspectives, and limitations

**DOI:** 10.3389/fphar.2026.1868595

**Published:** 2026-09-03

**Authors:** Alberto Orta, Miguel Angel Hidalgo-Leon, Carlos Lacalle-Gonzalez, Miriam M. Rodriguez de Lope, Lara Sanz-Criado, Jesus Garcia-Foncillas, Javier Martinez-Useros

**Affiliations:** 1 Medical Oncology Department, Hospital Clínic de Barcelona, University of Barcelona, Barcelona, Spain; 2 Translational Oncology Division, Comprehensive Cancer Center, Health Research Institute-Fundación Jiménez Díaz University Hospital, Universidad Autónoma de Madrid (IIS-FJD, UAM), Madrid, Spain; 3 START Madrid-FJD, Hospital Fundacion Jimenez Diaz, Madrid, Spain; 4 Area of Physiology, Department of Basic Health Sciences, Faculty of Health Sciences, Rey Juan Carlos University, Madrid, Spain

**Keywords:** apigenin, cannabinoids, curcumin, pancreatic cancer, plant-based drugs, quercetin, resveratrol, vitamins

## Abstract

Pancreatic cancer remains one of the most aggressive and lethal malignancies, with poor survival outcomes and limited therapeutic options. Surgical resection is currently the only potentially curative treatment; however, fewer than 20% of patients are eligible for this at diagnosis due to the stage of the advanced disease. Despite substantial progress in understanding the molecular pathogenesis of pancreatic cancer, effective targeted therapies remain scarce, and current systemic treatments provide only modest clinical benefit. In this context, natural compounds have attracted growing interest as potential anti-cancer agents because of their antioxidant, anti-inflammatory, and multi-target biological activities. Increasing preclinical evidence has demonstrated significant anti-tumor effects in pancreatic cancer models, including the inhibition of proliferation, induction of apoptosis, modulation of metastatic behavior, and enhancement of chemosensitivity. However, their clinical translation is still limited by poor bioavailability, pharmacokinetic variability, and incompletely characterized pleiotropic mechanisms of action. This review provides a comprehensive and critical overview of the current evidence regarding selected natural compounds in pancreatic cancer, including apigenin, cannabinoids, curcumin, luteolin, quercetin, resveratrol, vitamin C, and vitamin D. We summarize their molecular targets, therapeutic potential, and translational limitations, with the aim of supporting the design of future clinical studies and facilitating their integration into precision oncology strategies.

## 1 Introduction

Pancreatic cancer (PC) is the third leading cause of cancer-related mortality in both sexes. The annual number of cases closely mirrors the number of deaths, with 496,000 diagnoses and 466,000 deaths, making PC the deadliest type of cancer (Siegel et al., 2024). Its incidence in humans has increased in developed countries over the last decade and is expected to increase by 2030 to be the second leading cause of cancer-related deaths (Rahib et al., 2014). Tumor size at diagnosis is crucial for patient survival: a 5-year survival rate is around 50% for tumors < 2 cm, while 5-year survival rate could reach 100% when tumors are < 1 cm (Tamm et al., 2013). The main issue with PC is that it is asymptomatic in its early stages, and when symptoms do appear they are often misdiagnosed and commonly treated as outpatient, leading to late diagnosis. In those cases, the 5-year survival rate decreases to 3% (Kelsen et al., 1997; Siegel et al., 2019).

High levels of CA19-9 serum marker could serve as diagnostic biomarker (Duffy et al., 2010). However, this biomarker is not specific to PC, and biliary obstruction could increase its levels (Kim et al., 2004). Given this concern, the sensitivity of CA19-9 could be increased by up to 93.6% and its specificity to 95% for identifying PC patients in combination with serum levels of IGF-1 and albumin (Fe et al., 2016). Some of the most important risk factors associated with PC (Chen et al., 2015) are infection with *Helicobacter pylori* (65%), diabetes (30%) (Chari et al., 2008), smoking habits (20%–25%) (Bosetti et al., 2012; Blackford et al., 2009), and chronic pancreatitis (4%) (Lowenfels et al., 1997). Obesity, especially linked to high sugar, carbohydrate, red meat, and fat consumption and high alcohol intake, are also considered relevant risk factors (Kandaswami et al., 2005).

To date, surgical resection is the best approach against PC, and pathological features such as positive margins of resection (R1), affected regional lymph-nodes (N1), or tumor cells differentiation (G) could predict patient prognosis (Neoptolemos et al., 2018). However, due to the late-stage diagnosis characteristic of pancreatic cancer, only 15%–20% of patients qualify for this procedure. Even in cases of successful surgical resection, prognosis remains poor (Khorana et al., 2019). Various strategies have been investigated to enhance outcomes in patients with resectable PC, and adjuvant chemotherapy has emerged as one of the most effective solutions (Conroy et al., 2022). The CONKO-1 trial is the first to establish the advantages of adjuvant chemotherapy. Here, patients were randomized to receive either six cycles of gemcitabine at 1,250 mg/m^2^ (on days 1, 8, and 15 every 4 weeks) or observation. The study revealed a significant improvement in median progression-free survival (PFS) for the gemcitabine group (13.4 months; 95% CI: 11.4–15.3) compared with the control group (6.9 months; 95% CI: 6.1–7.8; p < 0.001), although it failed to demonstrate increased overall survival (OS) (Oettle et al., 2007). Subsequent trials, such as ESPAC-4 (Neoptolemos et al., 2020) and PRODIGE-24 (Conroy et al., 2018), explored alternative chemotherapy regimens to enhance patient outcomes and significantly influence the standard of care. The ESPAC-4 trial randomized resectable PC patients to receive gemcitabine monotherapy or a combination of gemcitabine and capecitabine for 6 months. This approach led to a 2.5-month improvement in OS. In the PRODIGE-24 trial, a modified 5-fluorouracil/leucovorin/irinotecan/oxaliplatin (FOLFIRINOX) regimen demonstrated a survival advantage of over 6 months compared with gemcitabine monotherapy. Consequently, FOLFIRINOX or gemcitabine/capecitabine have become the standard of care for patients with resectable PC. Although no head-to-head trials have directly compared these regimens, FOLFIRINOX appears to offer superior outcomes, and a comparative analysis has shown similar effectiveness (Orlandi et al., 2024). Recently, the NAPOLI-3 clinical trial has demonstrated that a combination therapy of liposomal irinotecan, 5-FU/leucovorin, and oxaliplatin (NALIRIFOX) outperformed gemcitabine plus nab-paclitaxel in terms of OS (11.1 months vs. 9.2 months) and PFS (7.4 months vs. 5.6 months) (Wainberg et al., 2023). Despite advances in the classic field of chemotherapy, research focused exclusively on PC has not led to significant breakthroughs in new oncological therapeutic areas. Targeted therapies based on immune therapy with anti-PD-1 for MSI-H/dMMR tumors or using targeted therapies against NTRK or RET when a fusion gene is present have shown some activity against PC (Maio et al., 2022; Berlin et al., 2020; Subbiah et al., 2022). However, this evidence comes from basket studies without a clear focus on these patients, and the frequency of these alterations in routine clinical practice is too low to achieve a great benefit. One recent studies on PC is the phase III POLO trial, which has shown a slight survival benefit in term of PFS without an OS benefit for maintenance with olaparib in patients with germline *BRCA1/2* mutations (Kindler et al., 2022). In early clinical trials, only sotorasib has emerged as a potential treatment option in phase I/II studies with *KRAS G12C* patients (Strickler et al., 2023). Recently, a new *KRAS G12*/ON multi-selective inhibitor (daraxonrasib) demonstrated clinically meaningful efficacy in a phase III randomized study (RASolute 302) in previously treated metastatic pancreatic ductal adenocarcinoma (Wolpin et al., 2026). Compared with standard-of-care chemotherapy, daraxonrasib significantly improved both overall and progression-free survival, establishing a novel targeted therapeutic strategy in *RAS-*driven disease. These results represent the first phase III evidence to support the direct pharmacologic inhibition of active KRAS signaling in advanced PC. Neoadjuvant treatment is used in borderline resectable tumors to make them resectable; it is commonly used in combinations such as FOLFIRINOX (folinic acid, 5-fluorouracil, irinotecan and oxaliplatin) or gemcitabine in with nano-albumin-bound paclitaxel (nab-paclitaxel) (Vera et al., 2016). For R1 tumors, borderline resectable, or locally advanced unresectable tumors, there is another option based on chemoradiotherapy (Mukherjee et al., 2013; Hammel et al., 2013). Nevertheless, PC is one of the most chemoresistant tumors due to a complex interaction between tumor cells and their microenvironment (Zeng et al., 2019). This microenvironment is composed of stromal cells such as myeloid derived suppressor cells (MDSCs), pancreatic stellate cells (PSCs), tumor-associated macrophages (TAMs), cancer-associated fibroblasts (CAFs), regulatory T cells (Tregs), and extracellular matrix (Ioannides and Whiteside, 1993). All these components promote hypoxia that provides not only proliferative and invasive capabilities but also chemoresistance and immune evasion (Apte et al., 2015; Chang et al., 2016). At the molecular level, PC exhibits a specific genomic profile characterized by mutations in *KRAS*, *CDKN2A*, *TP53*, and *DPC4* (Furukawa et al., 2006). Patients with a high familial component are associated with germline mutations in *ATM, BRCA2, MMR, PALB2, PRSS1*, or *STK11* (Rustgi, 2014). Although PC is genetically well defined, there are still no target therapies against this tumor.

Natural compounds present in plant-based products exhibit anti-tumor properties and have been used in traditional medicine for centuries to treat various diseases. Most of the positive effects of these compounds are due to their anti-oxidative and anti-inflammatory actions, which could be applied to treat different kinds of tumors. Oxidative stress, which is upregulated in tumors, promotes proliferation and chemoresistance. Additionally, several chemotherapies increase oxidative stress by generating reactive oxygen species (ROS). An increase in ROS can lead to cell death by ferroptosis, an iron-dependent and lipid-peroxidation-driven cell death cascade. Consequently, novel drugs are designed to target ROS and cell redox potential. In this context, natural compounds exhibit high anti-oxidative capabilities and open a broad range of new treatment approaches to overcome chemoresistance, metastasis, or serve as chemo-preventive agents. Importantly, all the natural compounds reviewed act on NRF2 and NF-κB, making them potential therapeutic agents against cancer. Some of these natural compounds are dietary polyphenols and have been extensively evaluated in cancer models as chemo-preventive agents (Thyagarajan et al., 2020).

Since PC lacks effective targeted therapies, natural compounds derived from plant sources have been extensively investigated for their potential therapeutic application, with several studies providing robust evidence to support their anti-tumor efficacy (Wang and Feng, 2015; Saha and Khuda-Bukhsh, 2013). Notably, these compounds are generally associated with minimal side effects, suggesting improved tolerability compared to many synthetic drugs commonly used in clinical practice. However, major limitations remain, including poor bioavailability, incompletely characterized mechanisms of action, and the absence of validated biomarkers to guide their clinical use (Rouse et al., 2014). Therefore, this review aims to critically synthesize the most relevant evidence on the therapeutic potential of natural compounds in pancreatic cancer while elucidating the molecular mechanisms underlying their activity and outlining future directions for their clinical translation.

## 2 Natural compounds: mechanisms of action and preclinical evidence

### 2.1 Apigenin

**FIGURE 1:**
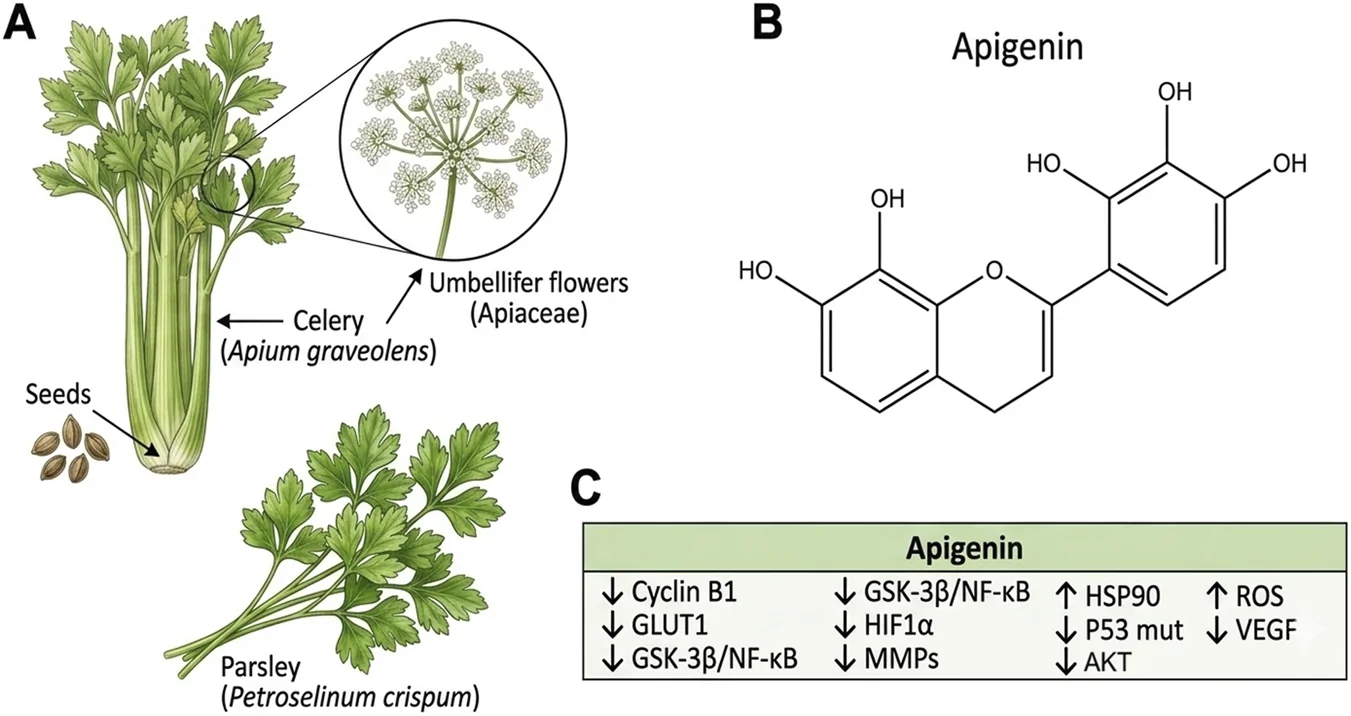
Molecular features of apigenin. **(A)** Apigenin is a bioactive flavonoid highly concentrated in the Apiaceae family, including celery and parsley. **(B)** Chemical representation of apigenin, showing the characteristic flavone backbone and hydroxyl group positioning essential for its biological activity. **(C)** Summary of key molecular alterations induced by apigenin. The compound downregulates (down-arrow) pro-survival and angiogenic factors (AKT, GLUT1, HIF-1alpha, VEGF, MMPs, cyclin B1, and mutant P53) and inhibits the GSK-3beta/NF-kB pathway. Conversely, it upregulates (up-arrow) HSP90 and promotes the accumulation of ROS, collectively driving apoptosis and suppressing tumor progression.

Apigenin (APG) (4′,5,7-trihydroxyflavone) is a flavonoid derived from the *Apium* genus of the Apiaceae family and is present in various vegetables, fruits, flavorings, and medicinal plants in warm tropical regions. Notably, celery and parsley are rich sources of apigenin (Figure 1A). The core structure of flavone compounds is the flavone nucleus, composed of two aromatic rings (A and B rings) linked by a three-carbon bridge (C ring), creating a C6-C3-C6 skeleton (Figure 1B). This compound shows several anti-inflammatory, antioxidant, and potential anti-tumor effects (Liu et al., 2024).

Several studies have evaluated the cytotoxic effects of apigenin in PC cell lines, demonstrating significant anti-proliferative activity in a dose-dependent manner, typically within the micromolar range (approximately 6–100 μM), depending on the cell model and experimental conditions (Ujiki et al., 2006). Apigenin is able to inhibit tumor cell proliferation and trigger apoptosis and cell cycle arrest. Moreover, it enables the inhibition of invasive phenotype through the downregulation of the AKT signaling pathway and matrix metalloproteinases (Figure 1C). Apigenin also reverts chemoresistance and blocks glucose transporter-1 (GLUT-1), hypoxia inducible factor (HIF), and vascular endothelial growth factor (VEGF) in pancreatic cancer cells at a dose of 50 μM (Melstrom et al., 2011). Such a dose enables the production of intracellular ROS in PC cells lines, which induce cytotoxicity in cell lines (Gilardini Montani et al., 2019). Another study with *in vivo* models reported an association with AKT modulation through SHIP-1 upregulation, which is a modulator of levels of phosphatidylinositol 3,4,5-trisphosphate, that promoted tumoricidal macrophages, enhanced anti-tumor immune responses, and reduced inflammatory factors in murine pancreatic cancer models, resulting in tumor shrinkage (Villalobos-Ayala et al., 2020).

The proposed underlying molecular mechanism of apigenin is through downregulation of the GSK-3β/NF kappa-B signaling pathway (Figure 1C). Apigenin was also able to arrest the cell cycle of PC derived cell lines at G2/M at 50 μM by downregulating cyclin B1. Furthermore, it has triggered the intrinsic apoptosis pathway at 50 μM and via the upregulation of apoptotic proteins and has increased levels of several cytokines such as IL17F, LTA, IL17C, IL17A, and IFNB1, supporting its anti-tumor activity (Johnson and de Mejia, 2013).

### 2.2 Cannabinoids

**FIGURE 2:**
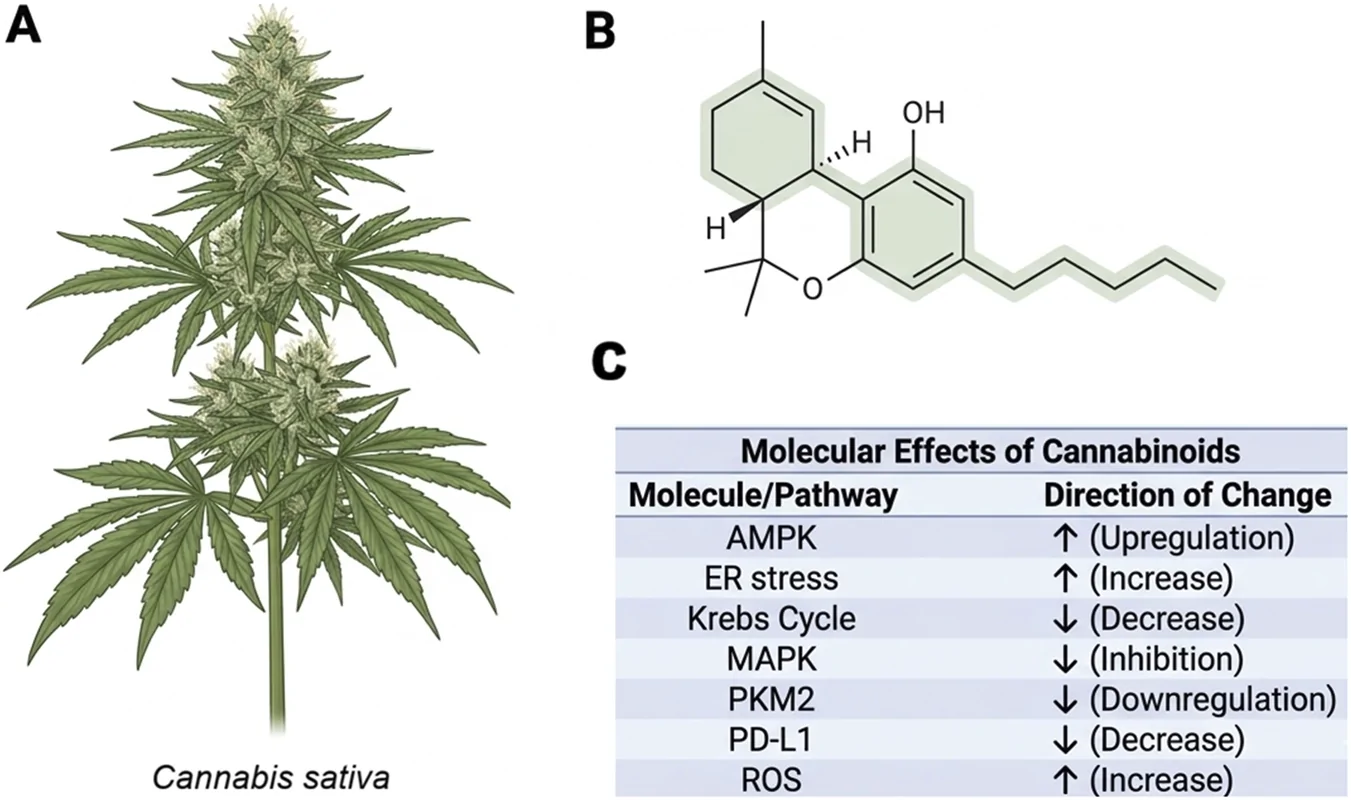
Molecular features of cannabinoids. **(A)** Cannabinoids are secondary metabolites derived from *Cannabis* plants. The female plants produce a highly lipophilic resin containing high concentrations of psychoactive terpenophenols. **(B)** Cannabinoids are structurally characterized by a unique C21-terpenophenolic backbone, as illustrated by the core scaffold essential for receptor binding and biological activity. **(C)** Summary of the metabolic and signaling alterations induced by cannabinoid exposure. Key effects include the upregulation (up-arrow) of AMPK, ER stress, and ROS production, alongside the downregulation or inhibition (down-arrow) of the Krebs cycle, MAPK signaling, PKM2 expression, and the immune checkpoint ligand PD-L1. These shifts contribute to the disruption of tumor homeostasis and promote antineoplastic effects.

Cannabinoids are produced by cannabis, which originated in Central Asia but is now grown worldwide. Cannabis female plants produce a highly fat-soluble resin that contains in a higher concentration than male plants of the psychoactive terpenophenol “cannabinoid” (Figure 2A). Cannabinoids are defined as a group of compounds that share a distinct C21 terpenophenolic backbone (Figure 2B). While they share this common structural origin, specific modifications in ring cyclization, oxidation states, and functional group arrangements give rise to their diverse chemical family (Radwan et al., 2021).

Cannabinoids exert their biological effects by modulating the endocannabinoid system (ECS), a complex network involved in maintaining cellular homeostasis. The primary mechanism involves retrograde signaling, where endocannabinoids are synthesized “on demand” and travel backward across the synapse to activate pre-synaptic CB1 and CB2 receptors. This leads to the inhibition of voltage-gated calcium channels and the suppression of neurotransmitter release, effectively acting as a neuromodulatory brake. While phytocannabinoids such as tetrahidrocannabinol (THC) directly bind to these receptors as agonists, others such as cannabidiol (CBD) function through non-canonical pathways, including the modulation of orphan receptors (GPR55) and ion channels, providing potent anti-inflammatory and neuroprotective effects (Lowe et al., 2021; Zou and Kumar, 2018).

Cannabinoid receptors CB1 and CB2 are found in the central nervous system, but the expression of CB2 is very low compared to CB1. The CB1 receptor is also found in peripheral nerve terminals (Kulkarni et al., 2017). Cannabinoids also have the ability to decrease pain symptoms through both ascending and descending pain pathways (King et al., 2017).

Concerning PC, the most studied cannabinoids are CBD and THC. Yang et al. (2020) found that these stopped both tumor cell proliferation and PSCs *in vitro* and in animal models. They reported that CBD and THC compounds carried out their role by directly acting through the MAPK pathway upon PC tumor cells and in PSCs via a P21 activated kinase 1 (PAK1).

Here, not only AMPK was upregulated by cannabinoid treatment but also an ROS-dependent increase of the AMP/ATP ratio. The reduction of pyruvate kinase isoform M2 (PKM2) reduced glycolysis and glutamine uptake and led to NADH accumulation, suggesting a respiratory chain arrest linked to reduced Krebs cycle activity (Figure 2; Dando et al., 2013). Some cannabinoids have achieved good anti-proliferative and pro-apoptotic results against PC tumor cells via activation of ATF-4 and TRB3 involved in endoplasmic reticulum stress (Carracedo et al., 2006). Moreover, a new cannabinoid derivative, FBL-03G, was evaluated with *in vivo* models, and not only tumors reduced after radiation therapy in combination with FBL-03G but survival was also longer and statistically significant compared to controls (*p* < 0.0001) (Moreau et al., 2019).

### 2.3 Curcumin

**FIGURE 3:**
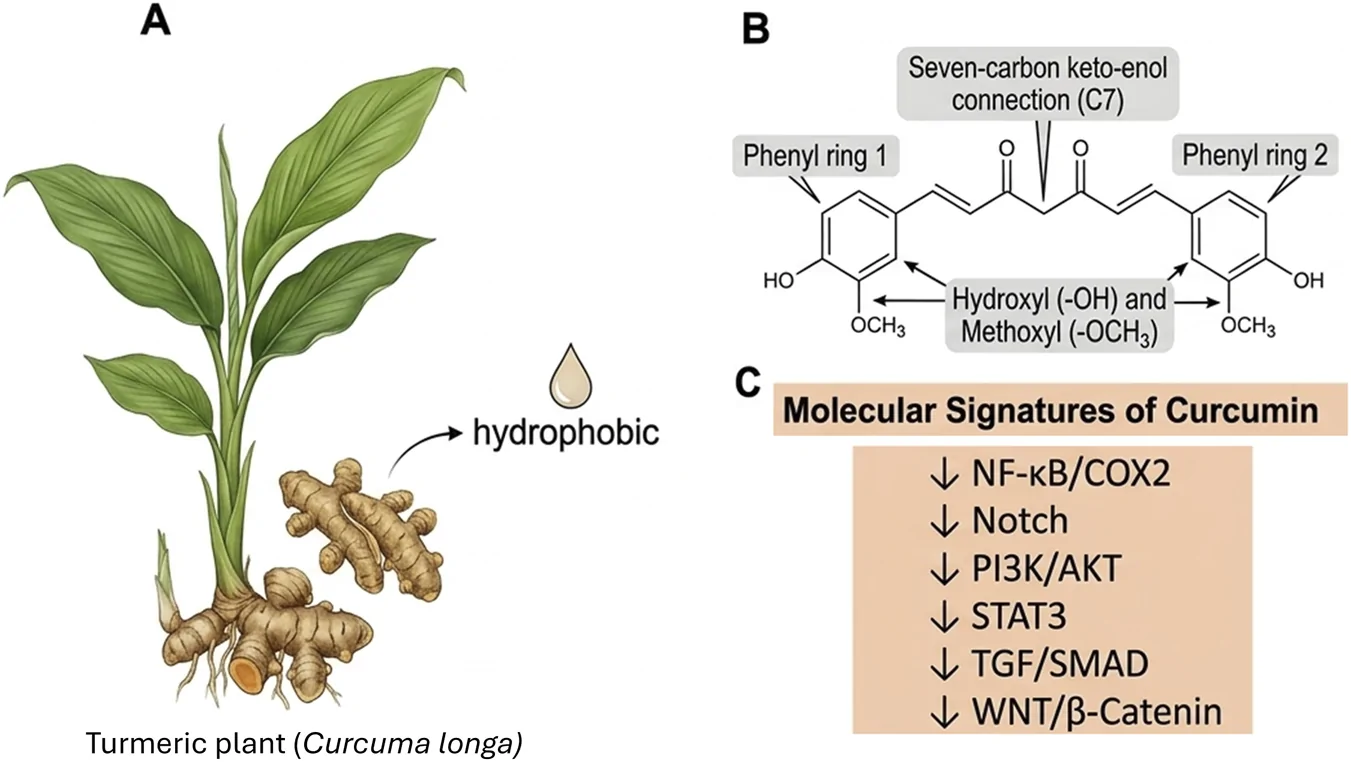
Molecular features of curcumin. **(A)** Curcumin is a hydrophobic, bioactive polyphenol extracted from the dried rhizomes of the turmeric plant, an herbaceous perennial of the ginger family. **(B)** Detailed chemical structure and key pharmacophores. The curcumin molecule is defined by two symmetrical phenyl rings connected by a seven-carbon (C7) flexible keto-enol tether. A key structural feature includes the symmetrical placement of distinct hydroxyl (-OH) and methoxyl (-OCH_3_) groups, which is critical for its antioxidant and biological activities. **(C)** High-purity curcumin exerts powerful, broad-spectrum downregulatory effects (down-arrow) across multiple interconnected oncogenic and inflammatory signaling networks. Key modulated pathways include NF-κB/COX2 (inflammation), notch (cell fate), PI3K/AKT, and STAT3 (proliferation and survival), TGF/SMAD (fibrosis and EMT), and WNT/β-catenin (stemness and invasiveness). The collective suppression of these pathways results in potent anti-proliferative, anti-invasive, and anti-inflammatory activity.

Curcumin is a hydrophobic polyphenol isolated from the dried rhizomes of turmeric (*Curcuma longa*) (Figure 3A). The chemical structure of curcumin consists of two phenyl rings joined by a seven‐carbon keto‐enol connection (C7) and replaced with hydroxyl and methoxyl groups (Figure 3B). The antioxidant activity of these molecules is predominantly ascribed to the 3′,4′-dihydroxy configuration (catechol group) situated on the phenolic B-ring, which represents the most potent radical-scavenging moiety within these compounds (Ahmadi et al., 2020). Although curcumin occurs naturally, most of its derivatives are synthesized by reacting aryl‐aldehydes with acetylacetone (Ahmad et al., 2024). This compound is largely used in traditional Chinese medicine; in addition, its antitumor potential has been reported in several kinds of cancer (Mansouri et al., 2020).

Curcumin is a highly pleiotropic molecule that exerts its biological effects through the simultaneous modulation of multiple signaling pathways (Figure 3C). Its primary mechanism involves the inhibition of the NF-κB transcription factor, leading to a significant downregulation of pro-inflammatory mediators such as COX-2, TNF-, and interleukins. In the context of oncology, curcumin disrupts the PI3K/AKT/mTOR and MAPK cascades, inducing cell cycle arrest and promoting mitochondrial-mediated apoptosis via caspase activation (Figure 3C). Furthermore, it enhances the cellular antioxidant response by upregulating the Nrf2/ARE pathway that effectively neutralizes ROS and maintains redox homeostasis (Esmaeli and Dehghanpour Dehabadi, 2025). Consequently, curcumin affects several factors and molecular pathways associated with tumorigenesis such as NOTCH, SHH, STAT3, TGF/SMAD, NF-κB/COX-2, and WNT/β-catenin (Figure 3C; Li et al., 2004; Celik et al., 2018).

Curcumin has also been investigated in several PC studies that demonstrate its biological activities of interest that support further evaluation of its potential role in pancreatic cancer. Curcumin has been found to induce apoptosis and inhibit cell growth and invasion *in vitro*, inhibit tumor growth and angiogenesis *in vivo*, and target cancer stem cells (Bimonte et al., 2013; Ning et al., 2016; Zhao et al., 2015; Ma et al., 2014). While curcumin has the ability to decrease the activity of both NF-κB and IkappaB kinase (Li et al., 2004), it increases FOXO1 expression levels (Zhao et al., 2015) in PC cell lines that affect proliferation and raise the apoptotic ratio. Ning et al. (2016) reported how curcumin attacks PC-derived cancer stem cells (CSC) more efficiently than their parental cell lines, suggesting that it could be a good treatment for eradicating this chemoresistant tumor population.

Curcumin demonstrates robust preclinical anti-tumor activity in pancreatic cancer models, including effects on apoptosis, angiogenesis, and key oncogenic pathways such as NF-κB and STAT3, as well as potential chemosensitizing properties.

### 2.4 Luteolin

**FIGURE 4:**
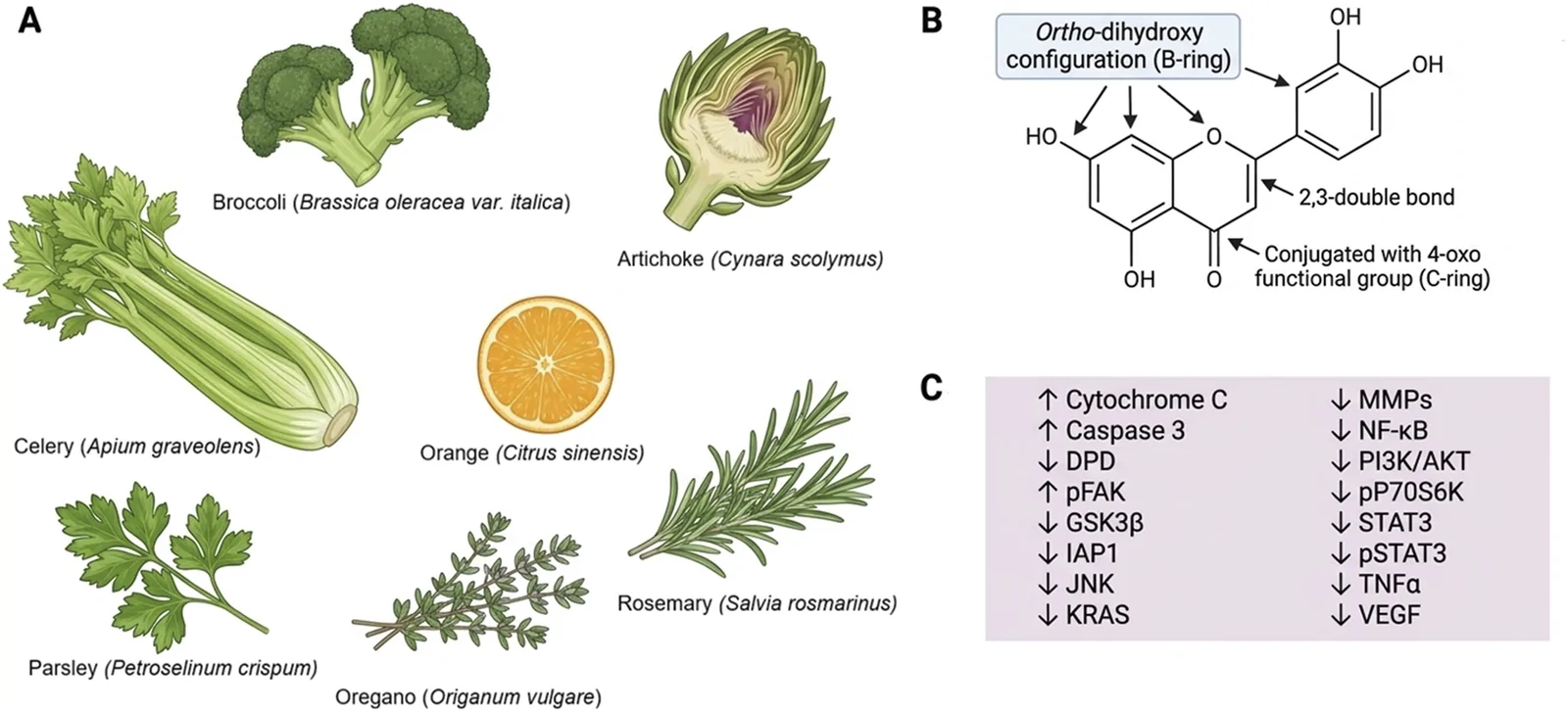
Antioxidant activity and molecular targets of luteolin. **(A)** Luteolin is a prevalent flavone found in a wide array of dietary sources, including vegetables such as celery, broccoli, artichoke, and carrots, as well as fruits such as oranges. It is also highly concentrated in culinary herbs, including parsley, thyme, rosemary, and oregano. **(B)** The potent antioxidant capacity of luteolin is pharmacologically attributed to specific structural features driven by the ortho-dihydroxy configuration (catechol group) in the B-ring and by the 2,3-double bond conjugated with the 4-oxo functional group in the C-ring. These elements facilitate electron stabilization and free radical scavenging. **(C)** Luteolin exerts comprehensive antineoplastic effects by upregulating (up-arrow) pro-apoptotic markers such as cytochrome C, caspase 3, and phospho-FAK (pFAK) while downregulating (down-arrow) key drivers of tumor progression, including KRAS, PI3K/AKT, STAT3, NF-κB, and angiogenic factors such as VEGF and matrix metalloproteinases (MMPs).

Luteolin is a prominent flavonoid distributed across various vegetables, medicinal herbs, and fruits. This molecule is widely found in many plants, including celery, broccoli, artichoke, oranges, green peppers, carrots, and olive oil, and some herbs such as parsley, thyme, dandelion, perilla, chamomile, peppermint, rosemary, and oregano (Figure 4A; Shimoi et al., 1998). Its significant antioxidant capacity is primarily attributed to the *ortho*-dihydroxy configuration of the B-ring and the 2,3-double bond conjugated with the 4-oxo functional group in the C-ring (Figure 4B; Imran et al., 2019). It has been demonstrated in both *in vitro* and *in vivo* models that luteolin is one of the most potent flavonoids that enable FAK phosphorylation and decrease the secretion of matrix metalloproteinases (Figure 4C; Kandaswami et al., 2005). Luteolin presents several therapeutic effects, such as anti-inflammatory and pro-apoptotic features, and can overcome chemoresistance in cancer cells. These observed effects are due to its ability to inhibit NF-kappaB by inhibiting TNF-alpha; furthermore, luteolin is able to downregulate the expression of NF- κB-related genes such as c-IAP1 and upregulate the expression of c-Jun N-terminal kinase (JNK) (Figure 4C; Shi et al., 2004). In terms of its antineoplastic potential, luteolin has demonstrated potent *in vitro* antiproliferative activity against diverse tumor cell lines, with values ranging 3–50 μM (Wruck et al., 2007). In murine xenograft models, it has arrested tumor growth and angiogenesis by inhibiting the VEGF-induced activation of AKT and phosphatidylinositol 3′-kinase (PI3K) activity, and it has inhibited VEGF-induced phosphorylation of P70S6 kinase (S6K) (Figure 4C; Bagli et al., 2004). Furthermore, *in vivo* studies have shown significant tumor growth inhibition when administered via dietary supplementation at concentrations of 50–200 ppm (Wruck et al., 2007).

In PC, luteolin was found to displace BAX from the hydrophobic cleft of BCL-2 that triggers mitochondrial apoptosis *in vitro* and inhibits tumor growth in xenograft models (Li et al., 2018). Luteolin has been evaluated followed by 5-fluorouracil or gemcitabine administration; however, decrease in cell viability was less than additive. Luteolin treatment combined with gemcitabine exhibited higher ratios of cell proliferation arrest. Huang et al. (2015) discovered that PC derived cell lines decreased their migration capability after luteolin supplementation through the regulation of epithelial-to-mesenchymal transition. Furthermore, they observed that luteolin downregulated, in a dose-dependent manner, matrix metalloproteases MMP2, MMP7, and MMP9 and deactivated the STAT3 signaling pathway.

### 2.5 Quercetin

**FIGURE 5:**
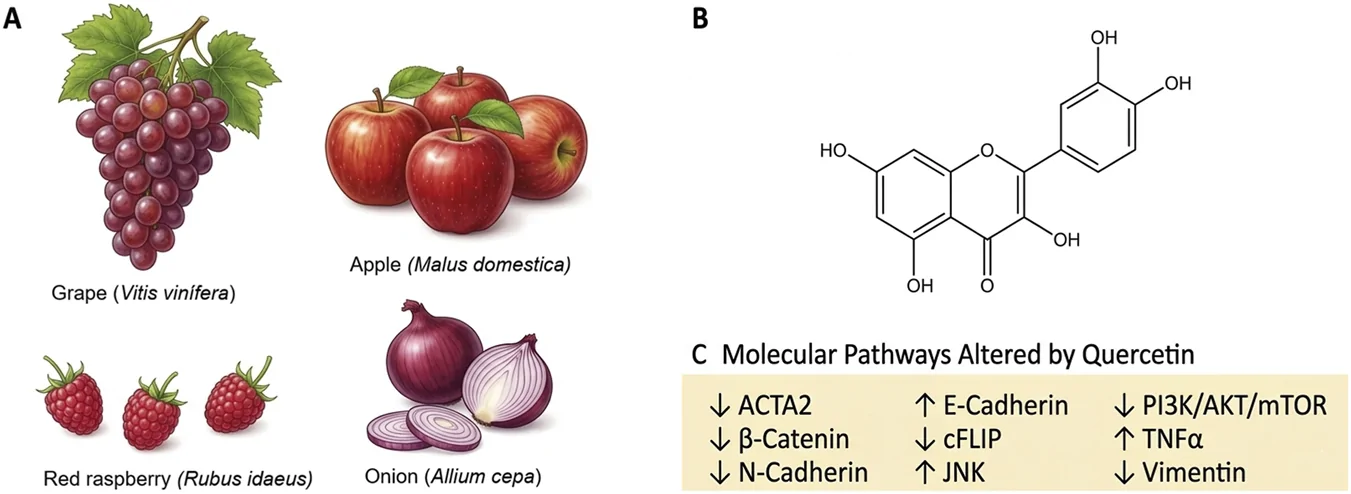
Sources, chemical structure, and signaling effects of quercetin. **(A)** Quercetin is a flavonoid found in a wide variety of grains, fruits, and vegetables. Rich sources include apples, red grapes, red raspberries, and onions. **(B)** The molecule is characterized by five hydroxyl groups positioned at carbon atoms 3, 5, 7, 3′, and 4′. This core structure serves as the basis for the formation of numerous quercetin glycosides. **(C)** Summary of the molecular targets and signaling pathways altered by quercetin treatment. Arrows indicate the direction of change, showing the downregulation (down-arrow) of markers associated with epithelial-to-mesenchymal transition (EMT) and pro-survival signaling and the upregulation (up-arrow) of factors involved in cell adhesion and stress response.

Quercetin is another flavonoid present in grains, many fruits such as apples, grapes, and red raspberries, and vegetables such as onions. This compound has shown diverse anticancer activities through several molecular mechanisms in preclinical models, highlighting its potential as a candidate for future studies in pancreatic cancer (Figure 5A). Quercetin serves as a multifaceted biological response modifier characterized by its potent antioxidant, anti-inflammatory, and senolytic properties. It is characterized by five hydroxyl groups at positions 3, 5, 7, 3′, and 4′. The core structure of this flavanol serves as the basis for several glycosides (Figure 5B). Notable examples include quercetin-3-O-glucoside (isoquercitrin) and quercetin-3-O-rutinoside (rutin), along with glycosylation occurring at the 4′ or 3,4′ positions (Liu et al., 2025). Its molecular mechanism primarily involves the direct neutralization of free radicals and the chelation of transition metals, complemented by the activation of the Nrf2/ARE antioxidant signaling pathway (Figure 5C). Furthermore, quercetin acts as a natural kinase inhibitor, disrupting the PI3K/AKT/mTOR signaling axis and inducing cell cycle arrest in malignant cells (Figure 5C). Notably, its ability to inhibit NF-κB and downstream pro-inflammatory enzymes, such as COX-2 and 5-LOX, positions it as a key regulator of the inflammatory microenvironment.

Kim et al. (2016) revealed that quercetin was able to induce chemosensibility in TRAIL-resistant PC cells with a subsequent apoptosis induction. Here, treatment with quercetin downregulated FLICE-like inhibitory protein (cFLIP) expression levels and activated JNK in a dose-dependent manner, which allowed proteasome degradation of cFLIP (Figure 5C) (Kim et al., 2016).

One of the mechanisms involved in the phenotypic switch by which cells lose their cell–cell adhesion and polarity and become highly migratory and invasive is epithelial-to-mesenchymal transition (EMT). In this regard, Hoca et al. (2020) reported how quercetin decreased expression levels of mesenchymal factors N-cadherin and ACTA-2 in cancer stem cells (Figure 5C). Yu et al. (2017) similarly found that quercetin can arrest EMT in PC cells *in vitro*. Here, E-cadherin was increased while vimentin and N-cadherin were decreased with increasing concentrations of quercetin (Figure 5C). Yu et al. (2017) also reported the inhibition of migration and invasion of PC cells by quercetin by the downregulation of matrix metalloproteinases MMP2 and MMP7, as well as the blockage of the phosphorylation of STAT3 in a dose dependent manner. These findings suggest that quercetin may interfere with metastatic-related mechanisms in PC cells, supporting further investigation of its potential role in pancreatic cancer progression. Another study demonstrated that quercetin disables the PI3K/AKT/mTOR signaling pathway to trigger apoptosis, autophagy, cell cycle arrest, and revert gemcitabine resistance in PC *in vitro* (Figure 5C) (Lan et al., 2019).

Nwaebu et al. (2016) found that miR let-7c was upregulated after quercetin treatment in two PC derived cell lines and in one primary tumor. Furthermore, let-7c was identified as targeting Numbl, an inhibitor of Notch, and functional experiments with a let-7c mimic decreased migration and stemness *in vitro*, which reduced tumor volume in xenograft models (Nwaebu et al., 2016). Subsequently, the same group identified and validated the miRNA expression profile induced by quercetin. Here, one of the miRNA they discovered, miR-200b-3p, was involved in the Notch-signaling/cell-fate determination; Numb, PROX1, TRIM2, and Notch1 exhibit putative binding sites of miR-200b-3p. Quercetin also significantly decreased tumorsphere formation, leading to a less aggressive tumor (Nwaeburu et al., 2017).

Quercetin can also target β-catenin in PC tumorspheres by inhibiting self-renewal capacity and the expression of their surface markers and decreasing their proliferation and invasion ability (Cao et al., 2015). A principal role of quercetin is the regulation of the Nrf2/SLC7A11/GPX4 axis that can promote ferroptosis; it thus provides another mechanism of action to the anticancer effect of this natural compound (Cruz-Gregorio and Aranda-Rivera, 2023; Xie et al., 2022).

### 2.6 Resveratrol

**FIGURE 6:**
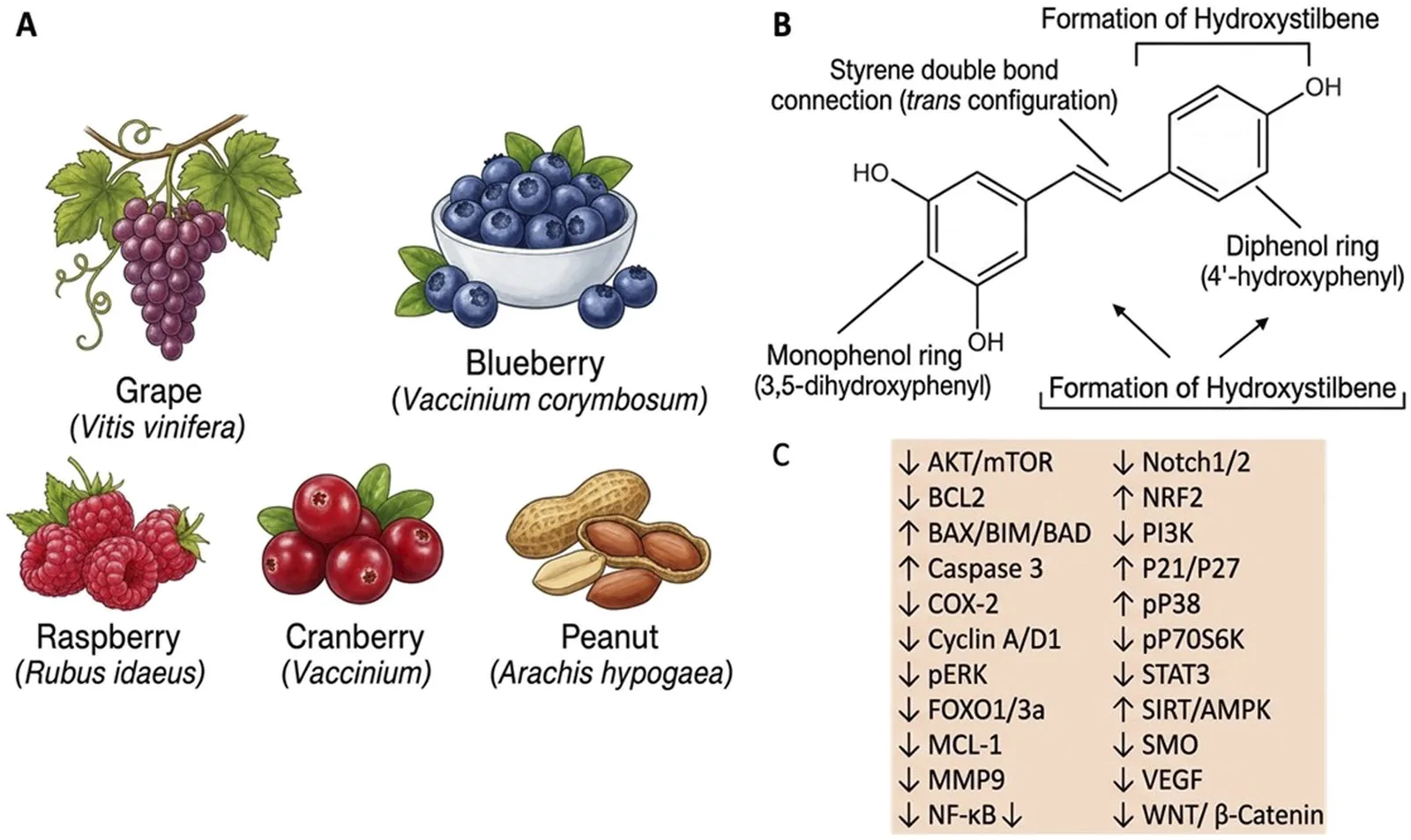
Natural sources, chemical structure, and signaling effects of resveratrol. **(A)** Resveratrol is a naturally occurring polyphenol found in various dietary sources, most notably in the skin of grapes, blueberries, raspberries, cranberries, and peanuts. **(B)** Resveratrol is a stilbene derivative synthesized through the hydroxylation of stilbene. Its structure is defined by two phenolic rings: a monophenol (3,5-dihydroxyphenyl) and a diphenol (4′-hydroxyphenyl). **(C)** Resveratrol exerts its antineoplastic and cytoprotective effects by influencing a broad range of pathways. It promotes apoptosis and cell cycle arrest by upregulating (up-arrow) BAX/BIM/BAD, caspase 3, p21/p27, and NRF2 while activating the SIRT/AMPK energy-sensing axis. Simultaneously, it downregulates (down-arrow) key oncogenic drivers, including AKT/mTOR, NF-κB, JAK/STAT3, WNT/β-catenin, Notch, and angiogenic factors such as VEGF.

Resveratrol is found in grape skin, blueberries, raspberries, cranberries, and peanuts, but most of the population consumes it in dietary supplements (Figure 6A). Resveratrol is a natural non-flavonoid phytoalexin and polyphenol formed from the hydroxylation of stilbene. It is thus a stilbene derivative characterized by two phenolic rings, a monophenol, and a diphenol, connected by a styrene double bond (Figure 6B). Naturally occurring in both *cis* and *trans* configurations, the trans isomer is recognized as the more prevalent and chemically stable form. The biological efficacy of the molecule is largely attributed to its three hydroxyl groups, which are instrumental in free radical quenching and metal chelation, while also mediating critical interactions with cellular macromolecules (Figure 6B; Warias et al., 2025).

Most studies have shown the anti-oxidative properties of resveratrol; however, few reports exist concerning its bioavailability, safety, and tolerability. Furthermore, although several studies have shown its anti-tumor features and its potential use in combination with other chemotherapies, the exact molecular mechanism of resveratrol remains unclear (Ko et al., 2017). Resveratrol presents anti-oxidative, anti-mutagen, and anti-inflammatory capabilities that act in all phases of tumorigenesis, from the inhibition of tumor initiation to the delay of tumor progression, growth, and metastasis (Ko et al., 2017).

Resveratrol influences a broad range of signaling pathways such as AKT/mTOR (Ge et al., 2013), Notch-1 (Zhang et al., 2014), Sirt1/AMPK and Nrf2 (Tamaki et al., 2014), STAT3 (Zhang et al., 2014), and WNT (Figure 6C; Zhang et al., 2014; Zou et al., 2015). Resveratrol is able to trigger apoptosis and autophagy in a dose dependent manner by downregulating anti-apoptotic proteins MCL-1 and BCL-2 and upregulating pro-apoptotic proteins BAX, BIM, BAD, and caspase-3 in tumor cells (Figure 6C) (Ge et al., 2013). Resveratrol induces cell cycle arrest through the upregulation of P21 and P27 and downregulation of cyclin A and D1 (Figure 6C). Interestingly, it also leads to the phosphorylation of P38-MAPK and the dephosphorylation of AKT, mTOR, and p70-S6K (Figure 6C; Ge et al., 2013). Zhang et al. (2014) found that resveratrol at 100 μM induced apoptosis through the downregulation of Notch1, Notch2, Hes1, STAT3 and Wnt2, Wnt5a, β-catenin, and phospho-STAT3 (Figure 6C).

Several studies support the anticancer activity of resveratrol in PC models, suggesting its potential as a candidate for further investigation. Qin et al. (2014) showed how resveratrol can inhibit tumor cell proliferation and induce apoptosis in *in vitro* models in a dose-dependent manner by downregulating the levels of Ihh, Ptch, and Smo1 (Qin et al., 2014). In PC, resveratrol induces cell proliferation arresting by the upregulation of P21 and P27 and the downregulation of Cyclin D1; it triggered apoptosis through the activation of Caspase-3. Resveratrol has also significantly decreased tumor growth from orthotopic PC models through the dephosphorylation of ERK, PI3K, AKT, FOXO1, and FOXO3a (Figure 6C; Roy et al., 2011).

### 2.7 Vitamin C

**FIGURE 7:**
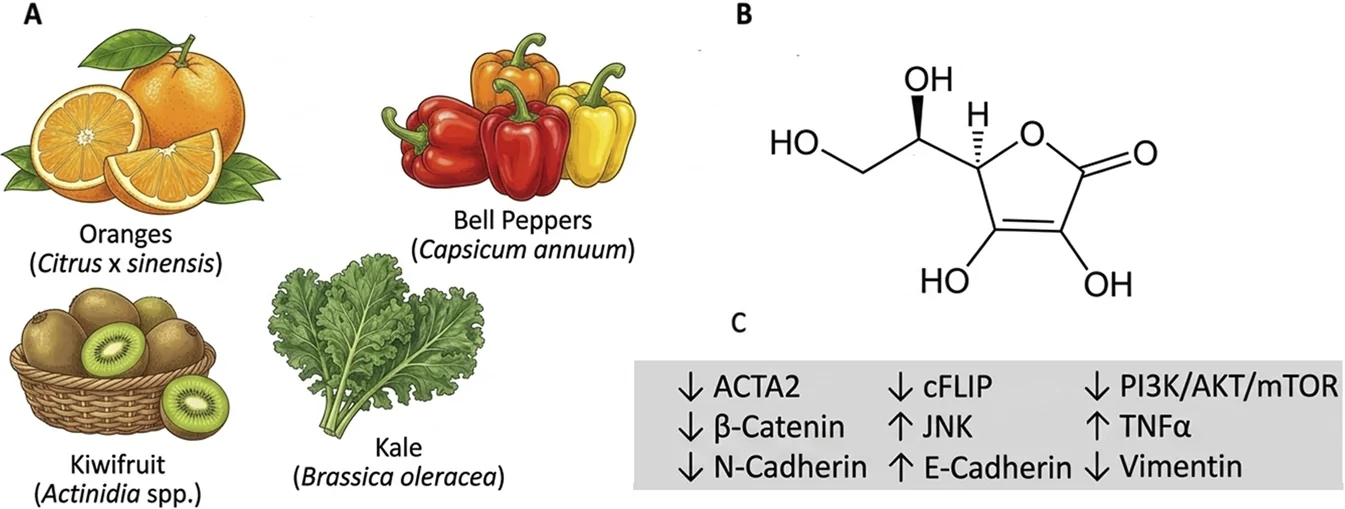
Dietary sources, chemical properties, and molecular modulation by Vitamin C. **(A)** Vitamin C is highly abundant in a variety of fruits and vegetables. Notable high-concentration sources include citrus fruits such as oranges, bell peppers, kiwifruit, and cruciferous vegetables such as kale. **(B)** Chemical structure of ascorbic acid: Vitamin C (C_6_H_8_O_6_) is a low-molecular-weight carbohydrate. Structurally, it is an analog of hexose sugars, featuring a distinct six-carbon lactone ring framework that enables its function as a potent reducing agent and antioxidant. **(C)** Summary of the principal molecular dysregulations induced by Vitamin C in a cancer context. Arrows indicate the direction of change, including the downregulation (down-arrow) of growth factor signaling (EGF/EGFR) and metabolic enzymes involved in glycolysis (GAPDH, GLUT1, PKM2). Additionally, it inhibits pro-survival signaling through the MAPK/ERK and PI3K/AKT pathways while promoting a pro-oxidant environment via the upregulation (up-arrow) of reactive oxygen species (ROS) in specific tumor environments.

Vitamin C (Vit. C) is mainly found in fruits and vegetables such as oranges, kiwi, strawberries, bell peppers, broccoli, and leafy greens. It is also present in smaller amounts in fortified foods and supplements. Because it is water-soluble and heat-sensitive, it is best obtained from fresh or lightly cooked foods (Figure 7A). Vit. C (C_6_H_8_O_6_), or ascorbic acid, is a low-molecular-weight carbohydrate structurally analogous to hexose sugars, characterized by its six-carbon framework. While highly soluble in water, it functions as a weak and inherently unstable organic acid. Due to its chemical nature, it is susceptible to oxidation, particularly when exposed to elevated temperatures, light, oxygen, alkaline conditions, or high-humidity environments (Figure 7B) (Alberts et al., 2025).

Vit. C is vital in many biological processes, particularly as an essential enzyme cofactor for dioxygenases and monooxygenases. This role is closely related to the synthesis of collagen and carnitine, the metabolism of tyrosine, and the regulation of gene transcription and translation. Specifically, Vit. C maintains transition metals such as Fe^2+^ and Cu^2+^ in their reduced states, preventing enzyme inactivation. Beyond these physiological functions, Vit. C also acts as a key factor for ketoglutarate dioxygenases, enhancing the activity of 10–11 translocation (TET) DNA hydroxylases and Jumonji-C-domain-containing histone demethylases (JHDMs). These enzymes are responsible for DNA and histone demethylation, which facilitates epigenetic reprogramming and DNA repair (Alberts et al., 2025).

However, at high pharmacological concentrations, this biochemical role shifts from a purely reductive function to a potent pro-oxidant activity. This mechanism of Vit. C is mediated by the production of extracellular hydrogen peroxide (H_2_O_2_), which diffuses into cells, overwhelming their antioxidant defenses and inducing oxidative stress that ultimately leads to cell death (Mussa et al., 2025).

Vit. C has been reported to target the CSC energy-hampering metabolism through the mitochondrial tricarboxylic acid cycle and oxidative phosphorylation, since this stem cell metabolism is based on oxidative phosphorylation instead of glycolysis. Therefore, Vit. C may represent a potential approach to modulate CSC activity (Figure 7C) (Bonuccelli et al., 2017).

Similar results were observed after treatment with Vit. C to *in vitro* and *in vivo*. Here, tumor cells showed an increased cell cycle arrest in G0/G1, apoptosis ratio, intracellular ROS levels, and higher tumor shrinkage than controls. Interestingly, gene analysis revealed that Vit. C could modify the expression of factors related to insulin receptor signaling, metabolism, oxidative stress, and mitochondrial respiration (Zhang et al., 2019).

Du et al. (2010) showed that Vit. C decreased cell viability and increased apoptosis independently of the caspases cascade of BxPC-3 and PANC-1 human PC-derived cell lines *in vitro* but had no effect on a non-tumor immortalized pancreatic cell line. *In vivo* experiments with Vit. C not only exhibited a very small tumor volume compared to control (138 mm^3^ vs. 472 mm^3^, respectively) but also increased the survival of mice in 10 days (*p* < 0.0001).

In contrast, Johnson and de Mejia (2013) evaluated different bioactive compounds from citrus fruit in human PC cells, and Vit. C exhibited the lowest effect, even at very high concentrations (>200 µM), compared to the other compounds; thus, it could be interesting to search for another citric compound.

### 2.8 Vitamin D

**FIGURE 8:**
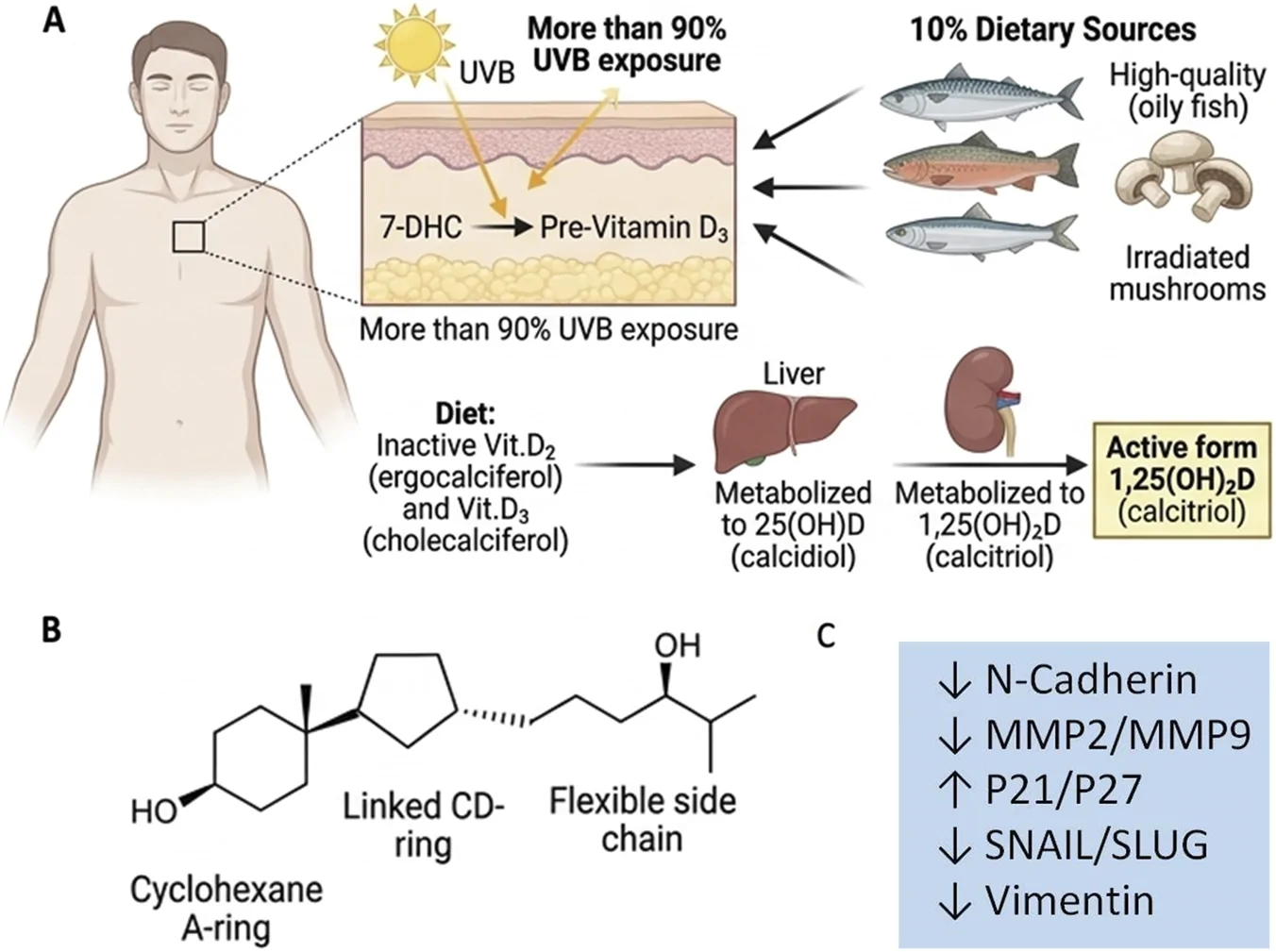
Overview of Vitamin D endogenous production, dietary metabolism, VDR interaction, and biological targets. **(A)** The majority (over 90%) of human Vitamin D is synthesized in the skin upon exposure to ultraviolet B (UVB) sunlight, where 7-dehydrocholesterol (7-DHC) is converted to pre-Vitamin D3. A smaller portion (10%) is obtained from the diet, specifically from oily fish (mackerel, salmon, sardines) and irradiated mushrooms. Dietary Vitamin D exists as inactive Vitamin D2 (ergocalciferol) and D3 (cholecalciferol). These forms must be sequentially metabolized, first by the liver to 25-hydroxyvitamin D [25(OH)D] (“calcidiol”), and subsequently by the kidney to the biologically active form, 1,25-dihydroxyvitamin D [1,25(OH)D_2_D] (calcitriol). **(B)** The active ligand 1,25(OH)D_2_D binds to the Vitamin D receptor (VDR) within a specialized binding pocket. The conformational dynamics of the cyclohexane A-ring are pivotal for this interaction. This unique conformational flexibility, linked via a conjugated triene bridge to the CD-ring system, facilitates the establishment of robust hydrogen bonds between the A-ring hydroxyl groups at the C-1 and C-3 positions and specific amino acid residues (e.g., Ser237, Tyr143, and His305, as detailed in the inset molecular view) within the VDR binding pocket. The biologically active forms 1,25(OH)D_2_D2 and 1,25(OH)D_2_D3 share a conserved structural architecture of the A-ring, linked CD-ring, and a flexible side-chain hydroxylated at C-25. **(C)** Active Vitamin D exerts pleiotropic effects by regulating key molecular targets involved in cancer cell invasion and proliferation. Arrows indicate the direction of molecular change: downregulation (down-arrow) of the cell adhesion molecule N-cadherin and the matrix-degrading enzymes MMP2/MMP9, and downregulation (down-arrow) of key epithelial-to-mesenchymal transition (EMT) transcription factors SNAIL/SLUG and the mesenchymal marker vimentin. Conversely, active Vitamin D upregulates (up-arrow) the expression of the cell cycle inhibitors P21/P27, thereby promoting cell cycle arrest.

More than 90% of Vitamin D (Vit. D) is obtained by humans from exposure to sunlight; the remaining 10% is obtained from the diet: oily fish such as mackerel, salmon, and sardines and from irradiated mushrooms (Figure 8A; Holick, 2004). From the diet, Vit. D is inactive as vitamins D2 (ergocalciferol) and D3 (cholecalciferol), and these forms needs to be firstly metabolized by the liver to 25-hydroxyvitamin D [25(OH)D], or “calcidiol,” followed by the kidney to 1,25-dihydroxyvitamin D [1,25(OH)2D], or “calcitriol” (Figure 8A). However, the use of calcitriol in clinical practice is limited due to its causing hypercalcemia (Lehmann and Meurer, 2010).

The biologically active forms of vitamin D 1, 25 (OH)_2_D_2_ and 1, 25 (OH)_2_D_3_, share a conserved structural architecture consisting of a cyclohexane A-ring, featuring hydroxyl groups at the C-1 and C-3 positions and a 19-CH_2_ methylene group at C-10, linked to a rigid CD-ring system via a conjugated triene bridge, and a flexible side chain hydroxylated at C-25 (Figure 8B). The conformational dynamics of the A-ring are pivotal for ligand-receptor interaction, facilitating the establishment of robust hydrogen bonds between the A-ring hydroxyl groups and specific amino acid residues within the Vitamin D receptor (VDR) binding pocket (Figure 8B; Powała et al., 2024). The mechanism of Vit. D is primarily defined by its role as a pro-hormone that undergoes sequential hydroxylation to its active form, which subsequently acts as a ligand for the VDR. Upon binding, the VDR forms a heterodimer with the retinoid X receptor (RXR) and translocates to the nucleus, where it binds to Vit. D response elements (VDREs) on the DNA to modulate the expression of hundreds of genes involved in calcium homeostasis, bone mineralization, and immune regulation. Additionally, Vit. D exerts rapid, non-genomic effects by interacting with membrane-bound receptors to activate intracellular signaling pathways, such as calcium channel modulation and kinase activation, providing a dual-action system for systemic and cellular regulation (Giustina et al., 2026; Quesada-Gomez and Bouillon, 2023). The National Institutes of Health (NIH) recommend serum levels Vit. D of 50 nmol/L or more; however, the Endocrine Society Clinical Guidelines Subcommittee recommend more than 75 nmol/L for the optimum development and metabolism of bone and muscle (Holick et al., 2011).

This compound has several anti-tumor effects through the modulation of cell cycle factors. One study with PC cells showed that Vit. D increased the expression level of P21 and P27 to arrest cell cycle in G1/S phase to induce growth inhibition (Figure 8C; Kawa et al., 1997). Interestingly, one derivative of Vit. D, 1,25-dihydroxyvitamin D(3)-3-bromoacetate, suppresses the *in vitro* proliferation of several PC-derived cell lines.

Schwartz et al. (2004) reported that both normal and PC tissues express high levels of 1-α-hydroxylase and VDR. This means that Vit D. could also be metabolized to the active hormone by the pancreas. Interestingly, their *in vivo* experiments revealed that dietary supplementation with cholecalciferol and calcium decreases the proliferation of pancreatic normal cells by arresting G1/S by the downregulation of P21/P27 (Figure 8C).

To prevent hypercalcemia due to Vit. D, a clinical-used variant, paricalcitol, binds VDR similarly to Vit.D. Interestingly, Martinez-Useros et al. (2021) reported that paricalcitol allows the unbundling of PC stroma. PSCs drive the production of the desmoplastic stroma and are particularly activated in PC. Interestingly, PSCs exhibit high expression levels of VDR, and treatment with paricalcitol inhibit the VDR of PSCs and hampers their stromal production (Apte and Wilson, 2012). Schwartz et al. (2008) showed that both Vit. D and paricalcitol inhibited PC cell proliferation and differentiation in a dose-dependent manner through the upregulation of P21 and P27. Moreover, paricalcitol decreased tumor growth of PC xenografts models without causing hypercalcemia (Schwartz et al., 2008). In colorectal cancer, Vit. D could induce ferroptosis, thus increasing ROS levels and decreasing cysteine and glutathione levels, leading to mitochondrial damage in colorectal cancer stem cells and thereby inducing ferroptosis. Additionally, the overexpression of SLC7A11 has reduced VD-induced ferroptosis, suggesting that VD promotes ferroptosis in CCSCs by downregulating SLC7A11. This offers new therapeutic insights for colorectal cancer (Guo et al., 2023).

Nevertheless, the biological rationale supporting stromal modulation and potential vascular normalization remains compelling, warranting further investigation in well-designed, biomarker-driven studies to better define patient subgroups that may derive clinical benefit.

### 2.9 Nimbolide

**FIGURE 9:**
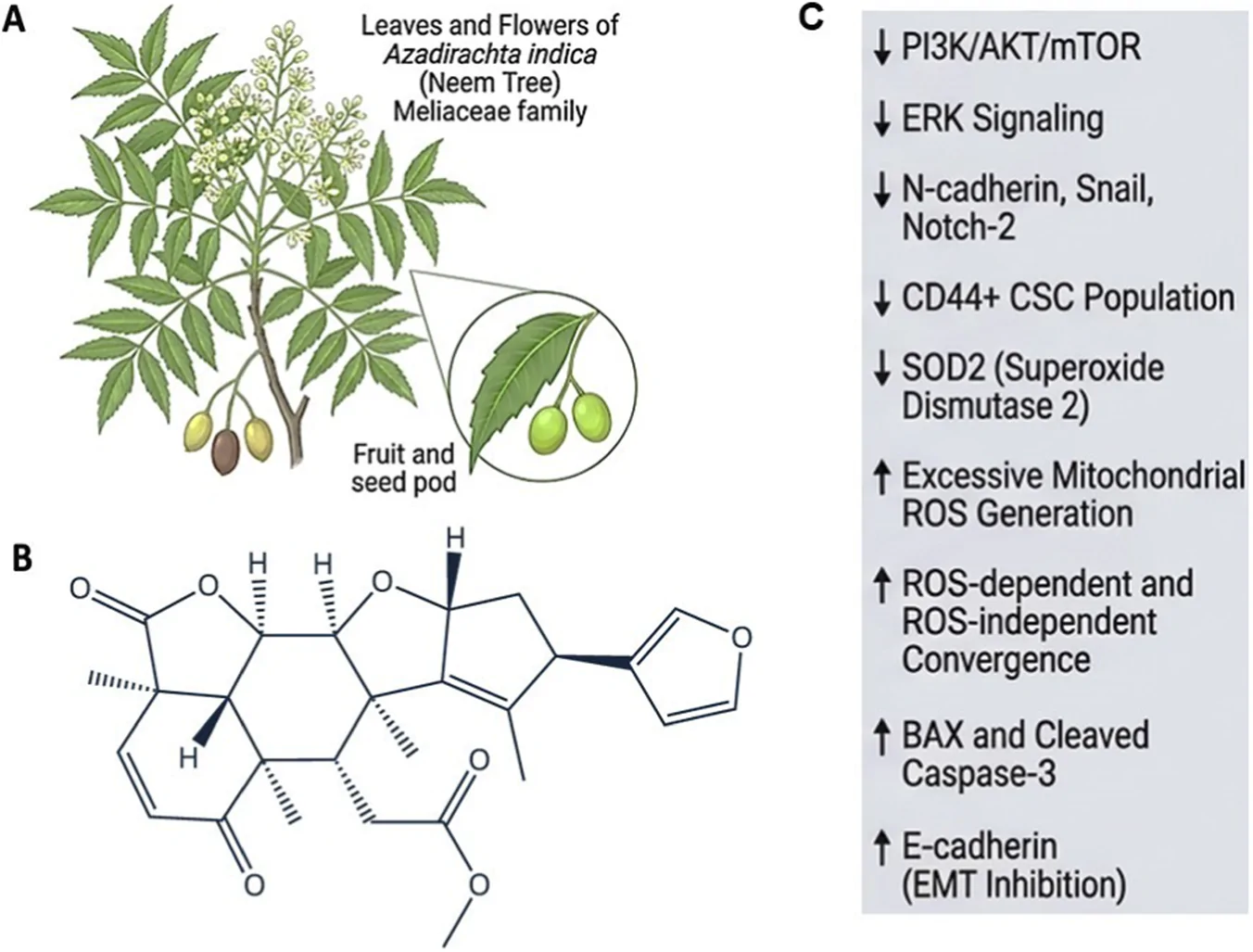
Natural source of nimbolide, chemical structure, and principal molecular mechanisms of action in cancer. **(A)** Botanical source of nimbolide, a bioactive limonoid isolated from the leaves, flowers, fruits, and seeds of *Azadirachta indica* (Neem tree; family Meliaceae). **(B)** The chemical structure of nimbolide is a tetranortriterpenoid limonoid characterized by a highly oxygenated polycyclic scaffold containing an α,β-unsaturated carbonyl moiety that contributes to its biological activity. **(C)** Schematic overview of the major molecular pathways modulated by nimbolide in cancer cells. Nimbolide suppresses oncogenic signaling by inhibiting the PI3K/AKT/mTOR and ERK pathways, downregulates epithelial–mesenchymal transition (EMT)-associated markers (N-cadherin, Snail, and Notch-2), reduces the CD44^+^ cancer stem cell (CSC) population, and decreases expression of the antioxidant enzyme superoxide dismutase 2 (SOD2). These effects promote excessive mitochondrial reactive oxygen species (ROS) accumulation, triggering both ROS-dependent and -independent apoptotic signaling pathways, including the activation of BAX and cleavage of caspase-3. Concurrently, nimbolide increases E-cadherin expression, thereby inhibiting EMT and reducing the invasive and metastatic potential of tumor cells.

Nimbolide is a limonoid terpenoid extracted from the leaves and flowers of the neem tree (*Azadirachta indica*, Meliaceae family) (Figure 9A). It presents a highly oxygenated limonoid-type terpenoid lactone characterized by a rigid tetranortriterpenoid scaffold and an electrophilic α,β-unsaturated lactone moiety. This structural framework confers unique three-dimensional features and enables interactions with key molecular targets through both covalent and non-covalent mechanisms. That confers potent electrophilicity, which underlies its covalent interaction with cysteine residues in target proteins (Figure 9B). Considerable research has focused on this phytochemical due to its anticancer potential. It inhibits the proliferation of various cancer cell lines and has exhibited therapeutic efficacy in preclinical studies (Jogi et al., 2025). In PC, nimbolide exerts potent antitumor activity through a convergence of ROS-dependent and -independent mechanisms. Subramani et al. (2016) demonstrated that it induces excessive mitochondrial ROS generation in PC cell lines, triggering mitochondrial-mediated apoptosis via the activation of BAX and cleaved caspase-3 while concomitantly suppressing PI3K/AKT/mTOR and ERK signaling to arrest proliferation. Critically, EMT was inhibited through the upregulation of E-cadherin and downregulation of N-cadherin, Snail, and Notch-2, with these effects confirmed in xenograft models (Figure 9C; Subramani et al., 2016). Other results focused on the selective reduction of the CD44-positive cancer stem cell population in PC and inducement of mitochondrial apoptosis in a dose-dependent manner, suggesting a capacity to target the chemoresistant CSC compartment that is responsible for tumor relapse and therapeutic failure (Kumar et al., 2018). Recently, it has been reported that nimbolide controls PC growth and metastasis by inhibiting superoxide dismutase 2 (SOD2), disrupting the antioxidant balance of tumor cells and thereby enhancing their susceptibility to oxidative cell death (Figure 9C; Mehmetoglu-Gurbuz et al., 2023). Perhaps the most mechanistically novel contribution is the identification by Li et al. (2023) of nimbolide as a covalent inhibitor of RNF114, a PARylation-dependent E3 ubiquitin ligase that normally drives PARP1 degradation at DNA lesion sites.

## 3 Clinical translation: evidence, trials and current limitations

### 3.1 Cannabinoids

Cannabis-based therapies have not yet received FDA approval for the treatment of cancer; consequently, clinical evidence supporting their use in oncology remains limited. However, some cannabinoids such as dronabinol and nabilone are commercially available for relief from the signs and symptoms of the adverse events of cancer or its treatments, for inducing sleep, and improving nutrition (Abrams, 2016). Beyond their supportive-care applications, emerging evidence suggests that cannabinoid-derived compounds may also possess direct antitumor activity. Cannabinoids have demonstrated improved survival compared to the placebo arm in other malignancies, such as glioblastoma or recurrent glioblastoma (Shimoi et al., 1998; Imran et al., 2019). Interestingly, FBL-03G, a non-psychoactive flavonoid derivative of cannabis, has been reported to modulate tumor-related pathways in preclinical models of PC, suggesting a possible avenue for future investigation. This compound has shown a strong ability to trigger apoptosis and proliferation arrest in combination with radiotherapy (Moreau et al., 2019). While definitive survival data for PC are currently pending following the completion of the Phase III DIsCOvER trial (NCT03984214) to evaluate oral THC in combination with FOLFIRINOX or gemcitabine/nab-paclitaxel, the preclinical evidence and established safety profile in supportive care suggest that cannabinoid-based therapies represent an adjunctive strategy that warrants further investigation (Supplementary Tables 1, 2; Arbeitsgemeinschaft medikamentoese Tumortherapie, 2025). From a translational perspective, cannabinoids represent a potential complementary approach to standard PC therapies, although further preclinical and clinical studies are required to establish their efficacy and safety in combination treatments, from simple palliative care to alleviating symptoms such as chemotherapy-induced nausea and vomiting severity and improving the quality of life of patients with advanced cancers to disrupt the tumor’s energy production through PKM2 inhibition and stripping its immunological “shield” via PD-L1 downregulation (Hardy et al., 2020; Good et al., 2019). This finding is of interest because PD-L1 downregulation in PC may reduce tumor-mediated immune evasion by limiting the ability of cancer cells to suppress T-cell activation through the PD-1/PD-L1 immune checkpoint pathway. While awaiting definitive DIsCOvER trial survival data, their established safety and synergistic potential with standard-of-care make them a compelling strategy for integrative oncology.

### 3.2 Curcumin

Curcumin has been evaluated in several clinical trials, the results of which are summarized in Supplementary Tables 1 and 2. In a clinical trial, 21 patients who were refractory to gemcitabine were treated with gemcitabine in combination with 8 g/day of curcumin. Interestingly, this combination treatment increased patient survival by 161 days (95% CI: 109–223 days) after 1 year, and the 1-year survival rate was 19% (4.4%–41.4%) compared to patients who received gemcitabine alone. Furthermore, oral curcumin was well tolerated by patients, with plasma levels of curcumin in plasma ranging from 29 to 412 ng/mL (Kanai et al., 2011). Other clinical studies have found that curcumin in combination with gemcitabine is better tolerated than FOLFIRINOX, although the latter exhibited better results in tumor control (Hosseini et al., 2017). Epelbaum et al. (2010) evaluated 8 g of curcumin orally/daily in combination with gemcitabine 1 g/m^2^ weekly for 3 of 4 weeks in 17 patients. Unfortunately, five patients discontinued treatment with curcumin due to abdominal pain, and in two other participants the dose of curcumin had to be reduced to 4 g/daily because of abdominal discomfort. At the end of this study, one patient (9%) presented partial response, four (36%) presented stable disease, and six patients (55%) presented tumor progression. Other studies suggest that the low toxicity profile of curcumin observed in patients may be related, at least in part, to its limited bioavailability. Here, the phase II clinical trial administered 8 g/day orally to 25 recruited patients, with only two achieving the appropriate biological activity (Dhillon et al., 2008). Only one patient presented with stable disease for longer than 18 months, and another presented with a tumor regression of 73% with an increased levels of IL-6, IL-8, IL-10, and IL-1. Dhillon et al. (2008) also reported how curcumin decreases, in peripheral blood mononuclear cells, the expression of NF-κB, COX-2, and the phosphorylated signal transducer and activator of transcription 3 (pSTAT3). Overall, the clinical evidence suggests that curcumin is a well-tolerated adjunctive compound with potential benefits in PC, particularly in combination with gemcitabine, although its antitumor efficacy remains limited and inconsistent. The major challenge for its clinical translation is its poor bioavailability, which may restrict the achievement of therapeutically relevant systemic concentrations. Therefore, optimized formulations and delivery strategies are required to fully evaluate the therapeutic potential of curcumin in pancreatic cancer.

### 3.3 Luteolin

A limited number of clinical trials involving luteolin or luteolin-containing formulations have been registered to date, targeting a heterogeneous range of conditions. These include neuropsychiatric disorders (such as schizophrenia, schizoaffective disorder, and autism spectrum disorders), metabolic diseases (notably type-2 diabetes), musculoskeletal conditions (including arthritis and myalgia), and various inflammatory or infectious indications such as COVID-19, allergic rhinoconjunctivitis, and oral mucositis. Notably, trial NCT03288298 investigated the potential apoptotic effects of a luteolin-based intervention, including both natural extracts and nanoparticle formulations, in models related to tongue squamous cell carcinoma; however, definitive clinical outcomes have not yet been reported (Supplementary Table 1). Luteolin in combination with chemotherapy was also evaluated in two clinical trials; in hepatocellular carcinoma, it prolonged overall survival and progression-free survival and reduced the mortality rate of patients (Yang et al., 2021); in ovarian cancer, a significant 34% decrease in incidence was observed for the highest versus lowest quintile of luteolin intake (RR = 0.66, 95% CI = 0.49–0.91; P = 0.01) (Gates et al., 2007). Unfortunately, no clinical trials have been conducted to date to evaluate luteolin in PC, highlighting a significant gap in its oncological clinical translation. Overall, current clinical research on luteolin remains fragmented, with heterogeneous endpoints, limited methodological standardization, and a scarcity of robust, peer-reviewed clinical evidence. Despite these limitations, ongoing exploratory studies provide a preliminary foundation for the future clinical evaluation of this flavonoid (Supplementary Table 2) (Elbaz, 2017).

### 3.4 Quercetin

Clinical evidence suggests that quercetin is a potent pleiotropic agent with diverse therapeutic applications. In inflammatory disorders, such as rheumatoid arthritis, daily doses of 500 mg significantly alleviate clinical symptoms, including morning stiffness and pain, and reduce plasma TNF-alpha levels (Javadi et al., 2017). Its metabolic benefits are equally notable, particularly in type-2 diabetes, where it improves antioxidant status and reduces serum concentrations of atherogenic oxidized LDL (Zohreh et al., 2014) as well as in PCOS (polycystic ovary syndrome), where it modulates androgenic profiles by reducing testosterone and LH levels (Rezvan et al., 2018; Rezvan et al., 2017). While the clinical data previously summarized highlights quercetin’s efficacy in metabolic and inflammatory diseases, its direct application in oncology remains fragmented. Clinical trials in prostate cancer (N = 60) have shown that doses of 1,000 mg/day can modulate oxidative stress markers and gene expression within tumor tissues (NCT01912820); however, therapeutic concentrations in the target tissue remain suboptimal for significant tumor regression (Jonsson Comprehensive Cancer Center, 2021). Early Phase I clinical trials in patients with various advanced cancers demonstrated that intravenous quercetin could inhibit tyrosine kinase activity *in vivo*; however, achieving therapeutic concentrations through oral administration remains a significant pharmacological challenge (Mayo Clinic, 2026). Concerning toxicity, quercetin was well tolerated by healthy subjects, although diarrhea was frequently observed (la Porte et al., 2010). Most recent clinical research has shifted toward the role of quercetin as a supportive adjuvant or as part of senolytic combinations, yet the absence of dedicated trials in PC is particularly noteworthy. This gap suggests that the clinical focus has been deterred by the molecule’s low bioavailability and the dense fibrotic stroma of PC, leaving a critical void in its investigation as a primary therapeutic scaffold for this malignancy.

### 3.5 Resveratrol

Resveratrol is able to regulate the insulin secretion of pancreatic β-cells under high glucose conditions and increase intracellular cAMP levels of β-cell lines and human islets (Rouse et al., 2014). In addition, in a phase I clinical trial, oral resveratrol was administered in 40 healthy volunteers, and no adverse event was observed. Although plasma levels achieved 539 ng/mL ( ± 384 ng/mL) at 1.5 h after the administration of two metabolites, monoglucuronides and resveratrol-3-sulfate levels were three- to eight-fold higher. Nevertheless, to achieve chemopreventive effects *in vitro*, concentrations of at least 5 μmol/L are required, which may be difficult to reach *in vivo* due to the extensive metabolism of resveratrol and the rapid formation of glucuronidated and sulfated metabolites. (Boocock et al., 2007). Resveratrol’s ability to regulate several factors and signaling pathways contribute to sensitizing PC cells to gemcitabine without increasing chemotoxicity (Gupta et al., 2011). Due to its ability to modulate mechanisms associated with chemotherapy response, resveratrol has been explored as a potential component of combination approaches with drugs such as gemcitabine, cisplatin, and 5-fluorouracil. Moreover, resveratrol appears to be well tolerated in patients, with preliminary evidence suggesting potential clinical and biological benefits at daily doses of 5 g or higher (Yiu et al., 2015). Overall, resveratrol represents a promising adjunctive strategy in PC due to its favorable safety profile and potential ability to enhance chemotherapy sensitivity. However, its limited bioavailability remains a major challenge that requires optimized formulations and further clinical validation.

### 3.6 Vitamin C

Several clinical trials have assessed the safety, tolerability, and potential efficacy of Vit. C in pancreatic cancer, although results remain rather heterogeneous (Supplementary Table 1). The efficacy of Vit. C in patients has been initially evaluated by the multiple Nobel Prize awardee Linus Pauling in 1976, who administered Vit. C at a dose of 10 g/day in combination with chemotherapy in 100 advanced cancer patients (13 stomach, 15 bronchus, 2 esophagus, 13 colon, 7 rectum, 6 ovary, 11 breast, 7 bladder, 2 gallbladder, 9 kidney, 2 lymphoma, 1 prostate, 1 uterus, 1 chondrosarcoma, 1 brain, 3 pancreas, 1 fibrosarcoma, 1 testicle, 1 pseudomyxoma, 1 carcinoid, 1 leiomyosarcoma, and 1 leukemia). The mean overall survival of those patients treated with Vit. C and standard chemotherapy was 210 days and, for the controls, only 50 days. Those patients presenting the highest responses had primary tumors located in the bronchus (*p* < 0.0001), colon (*p* < 0.003), stomach (*p* < 0.006), kidney (*p* < 0.002), rectum (*p* < 0.003), and ovary (*p* < 0.005) (*p*-value obtained with one-tailed analyses). Although these findings are intriguing, the clinical relevance of this study remains uncertain due to methodological limitations, particularly the small sample size within each tumor cohort, which restricts the statistical power and limits the generalizability of the results (Cameron and Pauling, 1976).

A phase I clinical trial with PC patients demonstrated that intravenous Vit. C was well tolerated, and the combination of Vit. C with gemcitabine achieved an overall survival 13 ± 2 months, while the median overall survival for gemcitabine-treated patients based on historic data was 5.65 months (Welsh et al., 2013). Vit. C intravenous was also evaluated in a phase I clinical trial with PC patients in combination with gemcitabine and an anti-EGFR (erlotinib). The reported safety data revealed no increased toxicity due to Vit. C supplementation to the combined treatment, and response rates concluded that 7/9 patients presented stable disease and 2/9 had progressive disease (NCT00954525) (Supplementary Table 1; Monti et al., 2012). Overall, clinical studies suggest that intravenous vitamin C may be a safe adjunctive strategy in PC, with preliminary evidence indicating potential benefits when combined with standard chemotherapy regimens. However, the heterogeneous study designs, small patient cohorts, and lack of randomized controlled trials limit definitive conclusions regarding its therapeutic efficacy.

### 3.7 Vitamin D

Cancer patients seem to have low levels of Vit. D, as demonstrated by Churilla et al. (2011), who measured Vit. D. levels of cancer patients from the same geographical region and found that 75% presented a significantly lower serum level of Vit. D (24.9 ng/mL) compared to non-cancer patients (30.6 ng/mL, *p* < 0.001). Furthermore, they also reported that low Vit. D levels are associated with advanced tumor stages. Concerning PC, Skinner et al. (2006) conduced a large-scale follow-up study with 46,771 men and 75,427 women to evaluate the association between Vit. D, calcium, and retinol intake with PC risk. Over 16 years, 365 individuals developed PC, and the study found an inverse correlation between Vit. D intake and PC risk but not with calcium or retinol. A Phase II single-arm trial involving 36 patients with inoperable PC demonstrated that high escalating doses of seocalcitol were well-tolerated, although no significant improvements in overall survival (OS) or time-to-treatment failure (TTF) were observed (Evans et al., 2002). Conversely, another Phase II study (n = 25) combining docetaxel with calcitriol for inoperable PC reported a modest extension in time to progression (TTP) compared to historical docetaxel monotherapy data. Interestingly, 3/25 patients exhibited partial response, 7/25 had stable disease, and a modest increased in time-to-progression was observed after combination treatment compared with historical data of docetaxel as a single agent (Blanke et al., 2009). More recently, an open-label pilot study (n = 20) explored paricalcitol in combination with nanoliposomal irinotecan and 5-FU/leucovorin for gemcitabine-resistant PC (NCT03883919) (Supplementary Table 1). While both tested regimens were safe, the weight-based dosing arm, which facilitated higher drug exposure, showed a notable increase in tumor vascularity (Grierson et al., 2023). Currently, numerous VDA-based clinical trials for PC remain registered on Clinicaltrials.gov, with several completed or ongoing studies awaiting final publication (Supplementary Table 1; Grosu et al., 2024). Overall, the clinical evidence evaluating Vit. D analogues in PC suggest an acceptable safety profile but only limited and inconsistent signals of antitumor efficacy when used in combination with standard chemotherapy regimens. While isolated studies have reported modest improvements in surrogate endpoints such as time-to-progression and changes in tumor vascularity, these findings have not yet translated into a reproducible survival benefit in larger or more definitive clinical trials. From a clinical perspective, Vit. D-based strategies should therefore not be considered part of standard-of-care treatment for PC, and their use should remain restricted to the context of well-designed clinical trials until more robust evidence of antitumor efficacy and survival benefit becomes available.

## 4 Strategies to overcome translational barriers

### 4.1 Apigenin

Despite its significant therapeutic potential, apigenin has yet to successfully transition into clinical trials for cancer treatment, primarily due to its stringent pharmacokinetic limitations (Supplementary Table 2). Its poor aqueous solubility and low intestinal permeability result in sub-therapeutic systemic bioavailability, while its rapid first-pass metabolism further prevents the molecule from reaching effective concentrations in target tissues, such as the pancreas (Shukla and Gupta, 2010). These challenges generate a translational gap in which the high doses required to replicate *in vitro* efficacy are clinically unfeasible. Consequently, the current paucity of active clinical trials underscores the urgent need for structural modifications and the synthesis of apigenin derivatives. Such strategies aim to optimize its lipophilicity and metabolic stability, providing a more viable scaffold for future human clinical evaluation (Cui et al., 2025). Overall, apigenin could be an anticancer compound, with its clinical translation currently limited by unfavorable pharmacokinetic properties rather than by a lack of biological activity. Future research focused on improving bioavailability and developing optimized derivatives will be essential to determine its therapeutic potential in pancreatic cancer.

### 4.2 Cannabinoids

Cannabinoids reduce neuropathy in animal models induced by treatment with cisplatin, paclitaxel, or vincristine (King et al., 2017). Interestingly, both CBD and THC have downregulated the expression of immune checkpoint PD-L1 in pancreatic tumor cells and in PSCs. These findings suggest that CBD and THC may exert immunomodulatory effects in PC and potentially enhance antitumor immune surveillance (Yang et al., 2020). Other studies propose using a combination of gemcitabine plus cannabinoid against CB1 and CB2 to inhibit PC growth. Here, PC-derived cell lines were treated and evaluated for AMPK activation. An increase in AMPK activity, induced by a ROS-dependent elevation of the AMP/ATP ratio, was observed, which subsequently promoted autophagy induction and inhibited cell growth (Dando et al., 2013). Gemcitabine presented a synergetic effect combined with cannabinoids in the inhibition of PC cell lines through ROS production, activation of the endoplasmic reticulum stress pathway, and induction of autophagic cell death (Donadelli et al., 2011). Overall, preclinical evidence suggests that cannabinoids may represent an efficient adjunctive strategy in PC by simultaneously modulating tumor immune escape, metabolic stress responses, and chemotherapy sensitivity. Their ability to enhance gemcitabine efficacy through ROS generation, AMPK activation, and autophagic cell death supports further investigation in translational and clinical studies.

### 4.3 Curcumin

Curcumin enables the sensitization of resistant PC cells to gemcitabine and arrests the growth of PC tumor xenografts in mouse models with combinational treatment (Yoshida et al., 2017). To improve curcumin bioavailability, a novel form of it has been formulated, achieving 27-fold bioavailability of curcumin in humans compared to curcumin powder (Sasaki et al., 2011). A phase I clinical trial evaluated the effect of this novel form of curcumin at two concentrations (200 mg (arm 1) and 400 mg (arm 2)) in combination with standard gemcitabine-based chemotherapy. Here, the plasma concentration of curcumin after administration of this novel form was between 324 ng/mL (range: 47–1,029 ng/mL) at arm 1 and 440/mL (range: 179–1,380 ng/mL) at arm 2. The most frequent adverse events were neutropenia, leucopenia, anemia, thrombocytopenia, and abdominal pain, and the major portion of the patients presented tumor progression (Kanai et al., 2013). However, clinical evidence remains limited, deriving mainly from small, early-phase studies that are underpowered and heterogeneous in design. While some trials suggest modest signals of tolerability and isolated clinical benefit in combination with gemcitabine, these findings are not consistently reproducible and lack confirmation in randomized controlled settings. A major translational barrier is curcumin’s poor systemic bioavailability, which makes it unlikely that *in vitro* effective concentrations are achieved in tumor tissue using standard formulations. Even with improved delivery systems, clinically meaningful outcomes in PC have not been convincingly demonstrated. Overall, curcumin should currently be regarded as an investigational adjunct with a strong mechanistic rationale but insufficient clinical evidence for therapeutic use in PC.

### 4.4 Luteolin

Molecular analyses have revealed that luteolin pretreatment followed by gemcitabine downregulates the expression levels of GSK-3β and NF-κB p65 and significantly upregulates the expression of cytochrome C to support its pro-apoptotic and anti-proliferative effects *in vitro* (Johnson and Gonzalez de Mejia, 2013). Other studies have evaluated the efficacy of luteolin in combination with gemcitabine in an orthotopic mouse model; this combination achieved a significant reduction in tumor volume (Johnson et al., 2015). This combination decreased expression levels of KRAS (46%, P = 0.0006), GSK-3β (34%, P = 0.014), phospho-GSK-3β (16%, P = 0.033), phospho-NF-κB(27%, P = 0.036), and BCL-2/BAX ratio (68%, P = 0.006). In contrast, the combination of treatment upregulated cytochrome C expression levels (44%, P = 0.035) and caspase 3 (417%, P = 0.003) (Johnson et al., 2015). Overall, these findings indicate that luteolin may enhance gemcitabine efficacy in PC by promoting apoptosis and suppressing key oncogenic signaling pathways. Although these preclinical results are interesting from a translational point of view, further studies are required to determine whether this combination can be translated into clinically meaningful benefits.

### 4.5 Quercetin

As with other flavonoids, quercetin therapeutic translation is frequently hampered by poor aqueous solubility and low systemic bioavailability (Alizadeh and Ebrahimzadeh, 2022; Gangwar et al., 2025). Quercetin has also achieved better results than gemcitabine alone in apoptosis induction in PC-derived cell lines. Moreover, in orthotopic *in vivo* models, both quercetin alone or in combination with gemcitabine showed a delay in tumor growth (Angst et al., 2013). Remarkably, the impact observed in Lan et al. (2019) after quercetin treatment was additive with gemcitabine effect, suggesting a potential therapeutic combination of quercetin plus gemcitabine. Therefore, the use of quercetin to target PC cells and decrease drug resistance in combination with gemcitabine is also supported in this study to improve the survival of PC patients (Cao et al., 2015). Overall, quercetin may contribute to the modulation of gemcitabine-induced apoptotic responses and delay tumor growth in preclinical models of PC. However, its limited bioavailability remains a major obstacle for clinical translation, highlighting the need for optimized formulations and further clinical evaluation.

### 4.6 Resveratrol

Zou et al. (2015) reported a reduction in cancer cell proliferation following resveratrol treatment, supporting its potential as a candidate for further investigation as an adjuvant approach in oncology. Harikumar et al. (2010) demonstrated that resveratrol could inhibit the proliferation of different PC-derived cell lines, leading to a synergetic effect in combination with gemcitabine. In orthotopic PC *in vivo* models, resveratrol exhibited a significant suppression of tumor growth that improved in combination with gemcitabine. Interestingly, the molecular studies of Harikumar et al. (2010) suggested that resveratrol hampers the activation of NF-κB and the expression of COX-2, cyclin D1, Bcl-2, Bcl-xL, MMP-9, and VEGF (Figure 6C).

KITC (N-hydroxy-N'-(3,4,5-trimethoxphenyl)-3,4,5-trimethoxy-benzamidine) is a synthetic polymethoxylated resveratrol derivative and has been reported as inducing apoptosis and inhibiting the growth of PC cell lines by a decrease in the activity of ribonucleotide reductase. This analogue inhibited cell growth synergistically in combination with gemcitabine *in vitro* (Bernhaus et al., 2009). The high inter-individual variability in its absorption, rapid hepatic metabolism leading to plasma clearance, short half-life (approximately 2–5 h), and limited bioavailability represent the main challenges associated with resveratrol that need to be addressed before its evaluation in clinical trials and potential implementation in the clinical management of PC (Yiu et al., 2015; Gambini et al., 2015). Overall, preclinical studies indicate that resveratrol and its derivatives can modulate key pathways involved in PC progression, including those related to proliferation, apoptosis, angiogenesis, and chemoresistance. However, despite encouraging preclinical evidence and synergistic effects with gemcitabine, pharmacokinetic limitations and poor bioavailability remain major barriers that must be overcome to enable successful clinical translation (Supplementary Table 2).

### 4.7 Vitamin C

In xenograft models carried out with seven different PC-derived cell lines, Vit. C was demonstrated to potentiate the anti-tumor effect of gemcitabine, and more than 50% tumor reduction was observed in combination than with gemcitabine alone (Espey et al., 2011). This dose-dependent effect seems to be associated with cytotoxic H_2_O_2_ production due to Vit. C catalysis. Overall, current evidence suggests that Vit. C may have greater therapeutic potential as an adjunctive strategy rather than as a standalone treatment, particularly by enhancing tumor sensitivity to conventional anticancer therapies. However, further clinical studies are required to validate these preclinical findings.

### 4.8 Vitamin D

When Vit. D is administered in combination with 5-amino-imidazole-4-carboxamide-1-beta-4-ribofuranoside (AICAR), the effect on proliferation is enhanced by 25%. This effect is observed by the ability of AICAR to induce the phosphorylation of AMPK, and the avoidance of AKT phosphorylation (Persons et al., 2010). A new generation, less calcemic analog of 1α,25(OH)_2_D3, 19-nor-2α-(3-hydroxypropyl)-1α,25-dihydroxyvitamin D3 (MART-10) caused a greater suppression of a PC-derived cell line tumor growth than the same dose of 1α,25(OH)_2_D3 without inducing hypercalcemia and weight loss (Chiang et al., 2013). These results were confirmed by the reversion of mesenchymal phenotype of tumor cells via the downregulation of Snail, Slug, vimentin and N-cadherin. Moreover, both drugs decreased MMP-2 and MMP-9 expression in the extracellular component (Chiang et al., 2014). These findings highlight the potential of Vit. D analogues and metabolic modulators as candidate therapeutic strategies in PC by targeting tumor proliferation, epithelial–mesenchymal transition, and invasive properties. The development of less calcemic analogues, such as MART-10, may overcome the limitations of conventional Vit. D compounds and support further clinical investigation.

### 4.9 Nimbolide

At the nanoformulation level, PLGA (poly-lactic-co-glycolic acid)-encapsulated nimbolide nanoparticles have been shown to induce mesenchymal-to-epithelial transition through dual AKT/mTOR inhibition in pancreatic CSCs *in vitro*, demonstrating the feasibility of nanodelivery approaches to overcome the compound’s inherent pharmacokinetic limitations (Supplementary Table 2; Singh et al., 2022). Despite this mechanistic richness, nimbolide is still in the early stages of drug development, and it currently lacks comprehensive pharmacokinetic characterization and long-term toxicity data *in vivo*. Moreover, no clinical trials have been initiated in PC or any other malignancy. Future research should prioritize formal pharmacokinetic profiling, establishing a safe dose range, and evaluating synergy with gemcitabine or PARP inhibitors in genetically stratified PC models, ideally in *BRCA-*mutant and *KRAS*-mutant contexts.

## 5 Towards precision oncology: biomarkers and patient stratification

### 5.1 Apigenin

Interestingly, treatment with apigenin decreased mTORC1 and mutant TP53 levels while increasing HSP90 expression. These findings suggest that apigenin may modulate antioxidant response through the mTOR–HSP90–mutTP53–p62–NRF2 axis, supporting further investigation of its potential to influence chemoresistance mechanisms in PC (Gilardini Montani et al., 2019). Apigenin also promoted P53 post-translational modification, translocation and DNA binding, upregulation of the P53-upregulated modulator of apoptosis (PUMA), and P21 proteins; *in vivo* models with *TP53* mutated PC-derived cell lines treated with apigenin presented smaller tumors than controls (King et al., 2012). These findings suggest that apigenin may represent a precision oncology approach in PC by targeting specific molecular vulnerabilities associated with *TP53* mutations and chemoresistance. The identification of predictive biomarkers, including *TP53* status and alterations within the mTOR-HSP90-NRF2 signaling axis, may enable patient stratification and improve the selection of individuals who could benefit from apigenin-based therapeutic strategies.

### 5.2 Luteolin

Pancreatic intraepithelial neoplasias (PanINs) are microscopic, non-invasive precursor lesions of pancreatic ductal adenocarcinoma (PDAC) that arise within the pancreatic ductal epithelium. They represent one of the most well-characterized pathways of PC development and are considered early stages in the multistep progression from normal pancreatic duct cells to invasive carcinomas. Recently, Kato et al. (2021) reported how luteolin suppressed the progression of PanIN to PC in animal models by reducing pSTAT3 and dihydropyrimidine dehydrogenase (DPD) expression. This raises the possibility that the luteolin-mediated suppression of DPD could represent a potential strategy to enhance 5-FU sensitivity in selected pancreatic cancer patients, although further clinical validation is required to determine whether DPD modulation can serve as a predictive biomarker for this combination therapy (van Kuilenburg, 2004). Overall, these findings highlight the potential of luteolin as a chemopreventive and therapeutic agent in PC by targeting early neoplastic progression and modulating clinically relevant pathways involved in treatment resistance. Luteolin’s ability to regulate DPD expression suggests a potential precision oncology strategy to identify patients who may benefit from 5-FU-based combination therapies.

### 5.3 Vitamin C

Tumors with catalase deficiency present higher toxicity to Vit. C. The higher the concentration of Vit. C, the higher the concentration of H_2_O_2_ in the tumor extracellular environment (Chen et al., 2007). In contrast, healthy tissues are able to generate enough catalase to protect themselves against these ROS and their toxic effects (Doskey et al., 2016). These findings support the potential of catalase expression as a predictive biomarker for patient stratification, and the selection of tumors that may be particularly susceptible to pharmacological Vit. C treatment. This approach exemplifies a precision oncology strategy by exploiting tumor-specific redox vulnerabilities while potentially preserving normal tissue integrity.

Vit. C treatment has also been demonstrated to eradicate *BRAF* wild-type tumor cells *in vivo* by activating ROS mechanisms and preventing the activation of EGF/EGFR-MAPK/ERK and PI3K/AKT pathways. Moreover, Vit. C was able to reduce *BRAF* mutant xenograft tumors by inducting the proteasome degradation of AKT and inhibiting ATP-dependent MAPK/ERK that support the role of Vit. C as a therapeutic supplement against thyroid cancer (Su et al., 2019). Yun et al. (2015) reported that an oxidized form of Vit. C (dehydroascorbate) achieved a higher cell-death ratio in cells carrying *KRAS* mutation than those not oxidized through GLUT1 and the inactivation of glyceraldehyde phosphate dehydrogenase, resulting in energy deficiency and cell death. Interestingly, they report how high levels of Vit. C could eradicate specifically human derived CRC cells lines *in vitro* and *in vivo,* not only harboring *KRAS* mutations but also *BRAF* mutations, compared to their non-mutant counterparts by an increase in oxidative stress (Yun et al., 2015). One year later, we published a study that confirmed their findings but focused on Vit. C and its interaction with the Warburg effect (Aguilera et al., 2016). In this study, we were able to dissect a novel molecular pathway of Vit. C that involved the disruption of the Warburg metabolism, specifically in *KRAS* mutant CRC. We observed in both *in vitro* and *in vivo* models derived from *KRAS* mutant cell lines an increased apoptotic ratio and a significant tumor reduction after Vit. C treatment, but not in normal colonocytes (Aguilera et al., 2016). A subsequent study from our group supported these previous results, in which Vit. C increased PDH activity compared to normoxic conditions and induced ATP depletion while increasing citrate synthase activity in *KRAS* mutant CRC-derived cell lines and murine xenografts (Cenigaonandia-Campillo et al., 2021). These results observed in CRC could also match other *KRAS* mutant malignancies, which is the case in PC that presents *KRAS* mutation in more than 90% of patients (Buscail et al., 2020). Di Tano et al. (2020) described *in vitro* and *in vivo* how Vit. C in combination with a fasting-mimicking diet increased the anti-tumor effect and potentiated oxaliplatin effects in *KRAS* mutated tumors such as CRC and PC and showed low toxicity via an increase in ROS. However, it is important to note that Vit. C administration may be contraindicated in patients with glucose-6-phosphate dehydrogenase (G6PD) deficiency due to the risk of hemolysis (Bigelse, 2018). These findings highlight the potential of Vit. C as a biomarker-driven therapeutic strategy in PC, where the high prevalence of *KRAS* mutations may represent an exploitable metabolic vulnerability. Future clinical studies that integrate molecular profiling, including KRAS status and redox-related biomarkers, will be essential to identify responsive patient subgroups and establish the role of Vit. C within precision oncology frameworks.

### 5.4 Vitamin D

It is remarkably that the Vit. D analogue MART-10 has exhibited both anti-proliferative and anti-migratory features in PC-derived cell lines independently of *KRAS* mutation (Chiang et al., 2014). Subsequently, treatment with 1-α,25(OH)_2_D3 and MART-10 could reduce PC proliferation in *KRAS* mutant cell lines in both *in vitro* and *in vivo* experiments, suggesting a potential treatment approach or a chemo-preventive drug against PC (Schwartz et al., 2004). Therefore, Vit. D analogues, particularly MART-10, may represent a good natural compound by targeting tumor proliferation and migration independently of *KRAS* mutational status. Further studies are needed to identify predictive biomarkers, such as Vit. D receptor signaling activity, that could enable patient stratification and optimize the clinical application of Vit. D-based therapies.

### 5.5 Nimbolide

Li et al. (2023) reported that nimbolide prevents PARP1 removal from damaged DNA by inhibiting RNF114 activity, resulting in profound PARP1 trapping and synthetic lethality selectively in *BRCA*-mutated cancer cells. This mechanism is of particular translational relevance in pancreatic cancer, where approximately 5%–10% of patients harbor germline *BRCA1/2* mutations and may represent a molecularly defined subgroup that is potentially susceptible to therapies that exploit homologous recombination deficiency, including future nimbolide-based combination strategies with PARP inhibitors. These results highlight nimbolide as a potential biomarker-driven therapeutic approach, especially for *BRCA-*mutated PC, by exploiting defects in homologous recombination repair. Further clinical investigation will be required to determine whether *BRCA* status can serve as a predictive biomarker for patient selection and guide the integration of nimbolide with PARP inhibition strategies.

## 6 Conclusions and future perspectives

The clinical translation of natural compounds in PC treatment is still significantly hindered by a complex interplay of pharmacological, biological, and pharmacokinetic limitations (Supplementary Table 2). Despite robust preclinical evidence that demonstrates the anti-tumor efficacy of agents such as apigenin, curcumin, luteolin, and quercetin, their integration into routine clinical practice is limited by suboptimal bioavailability, rapid systemic clearance, and a lack of standardized, weight-adjusted dosing regimens. As observed in multiple trials, the inherent structural complexity of many phytochemicals results in poor aqueous solubility and variable absorption, as exemplified by curcumin. These factors, coupled with their pleiotropic molecular mechanisms, complicate the establishment of clear dose-response relationships and the determination of optimal therapeutic windows (Bhajan et al., 2026).

To overcome these challenges, a paradigm shift toward rational drug design, including semi-synthetic derivatives and nanotechnology-based delivery systems, is essential. Synthetic analogs and nanoformulations, such as those derived from cannabinoids or polyphenolic scaffolds, are being engineered to enhance metabolic stability and facilitate drug delivery within the dense, desmoplastic stroma characteristic of PC, which currently acts as major physical and biochemical barrier against conventional therapies (Bhajan et al., 2026; Huang et al., 2021). By optimizing the chemical scaffolds of natural leads, it is possible to improve their “drug-likeness” and achieve more predictable pharmacokinetic and toxicity profiles (Paerhati et al., 2026). In this regard, targeting the glycolytic dependency of PC cells through the modulation of GLUT-1 or PKM2 using compounds such as Vit. C or apigenin may provide insights into strategies to overcome stromal resistance (Fakhri et al., 2024; Kim et al., 2022).

A major challenge in the clinical development of novel anticancer agents is the limited availability of validated pharmacodynamic (PD) biomarkers capable of monitoring biological activity and treatment response in real time. In this context, the evaluation of ferroptosis-related signatures, including GPX4 inhibition, 4-HNE-mediated lipid peroxidation, and intracellular redox alterations, may represent a valuable approach for assessing target engagement and optimizing dose selection in clinical settings (Yang et al., 2014; Park et al., 2025). Furthermore, investigating epigenetic alterations induced by these compounds, such as changes in histone acetylation and DNA methylation patterns, could provide deeper insights into their long-term mechanisms of antitumor activity and potential therapeutic applications (Duan et al., 2025).

Finally, the widespread availability of natural supplements as over-the-counter products represents a significant challenge due to uncontrolled consumption, variable product quality, and potential herb–drug interactions, which may confound clinical trial interpretation and compromise patient safety (Duan et al., 2025). The development of standardized and regulated formulations would enable accurate dosing, improve reproducibility, and facilitate rigorous clinical evaluation of these compounds, either as monotherapies or as potential synergistic partners with established treatment regimens such as FOLFIRINOX or gemcitabine/nab-paclitaxel (He et al., 2022). Ultimately, translating natural compounds into evidence-based oncological interventions will require multidisciplinary research that integrates pharmacology, biomarker validation, molecular stratification, and well-designed clinical trials to define their safety, efficacy, and appropriate therapeutic context.
