# Supplementary material for: Targeting pancreatic cancer with natural compounds: molecular mechanisms, preclinical evidence, clinical perspectives, and limitations
Source: Front Pharmacol. 2026 Sep 3;17:1868595. doi: 10.3389/fphar.2026.1868595 (PMC13582323; doi:10.3389/fphar.2026.1868595)
Supplement: Supplementary file 1 [file Table2.docx]

# Supplementary Table 2. Natural Compounds in Pancreatic Cancer: Molecular Targets, Preclinical Evidence, Clinical Translation and Pharmacokinetic Limitations

| **Compound** | **Principal Molecular Targets** | **Signaling Pathways** | **Preclinical Efficacy in PC** | **Clinical Evidence** | **Pharmacokinetic Limitations** | **Toxicity Profile** | **Clinical Development Stage** |
| --- | --- | --- | --- | --- | --- | --- | --- |
| **Apigenin** | AKT, GLUT-1, HIF-1α, VEGF, MMPs, GSK-3β, NF-κB, mTORC1, mutant TP53, HSP90, SHIP-1, Cyclin B1 | PI3K/AKT; GSK-3β/NF-κB; mTOR-HSP90-mutP53-p62-NRF2; VEGF/angiogenesis; G2/M cell cycle arrest | Inhibits proliferation (6–100 µM, dose-dependent); induces apoptosis via intrinsic pathway; blocks EMT and invasion; upregulates SHIP-1 → tumoricidal macrophages; generates intracellular ROS; reduces tumor volume in murine PDAC models | No clinical trials conducted in PC to date. Phase I/II gap unresolved due to pharmacokinetic barriers | Poor aqueous solubility; low intestinal permeability; extensive first-pass metabolism; sub-therapeutic systemic bioavailability; doses needed for efficacy clinically unfeasible | Generally well-tolerated at preclinical doses; no significant toxicity reported in vitro/in vivo | Preclinical only (no active PC clinical trials) |
| **Cannabinoids (CBD/THC/FBL-03G)** | CB1/CB2 receptors, PD-L1, MAPK/PAK1, PKM2, AMPK, ATF-4/TRB3 (ER stress), AMP/ATP ratio | Endocannabinoid system (CB1/CB2); MAPK/ERK; AMPK/autophagy; ER stress pathway; glycolysis inhibition (↓PKM2, ↓Krebs cycle); ROS-mediated cell death | CBD+THC inhibit tumor cell and PSC proliferation; downregulate PD-L1; FBL-03G + radiotherapy → significant tumor shrinkage and survival benefit (P<0.0001); gemcitabine + cannabinoid synergy via ROS/ER stress/autophagic death | Phase III DIsCOvER trial (NCT03984214) completed 2024 – survival data pending. Established palliative use (nausea, pain, QoL). Improved survival in glioblastoma models. | High lipophilicity; variable absorption; THC undergoes extensive hepatic metabolism; CBD bioavailability ~6% oral. Psychoactive effects limit dosing for THC | Dronabinol and nabilone approved and well-tolerated; psychoactive effects with THC; non-psychoactive FBL-03G shows favorable safety | Phase III (DIsCOvER/NCT03984214); palliative use approved; awaiting survival results in PC |
| **Curcumin** | NF-κB/IκB, PI3K/AKT/mTOR, COX-2, STAT3, FOXO1, EZH2, PVT1 (lncRNA), β-catenin, cancer stem cells | NF-κB/COX-2; PI3K/AKT/mTOR; MAPK; NOTCH; SHH; TGF-β/SMAD; WNT/β-catenin; Nrf2/ARE antioxidant axis; mitochondrial apoptosis (caspase cascade) | Induces apoptosis, inhibits proliferation/invasion; suppresses angiogenesis (in vivo); sensitises resistant cells to gemcitabine; targets CSC population more efficiently than parental lines | Phase I/II trials (n=17–25) with 8 g/day oral ± gemcitabine: modest signals (1 patient 73% regression; median OS 161 days); only 2/25 achieved biological activity. Novel Theracurmin form (27× bioavailability) tested in Phase I – most patients still progressed | Poor aqueous solubility; erratic absorption; rapid systemic clearance; plasma levels 29–412 ng/mL (standard); scarce bioavailability main barrier. Theracurmin improved to 324–440 ng/mL but insufficient for tumor penetration | Oral formulation generally well-tolerated; 5/17 patients discontinued due to abdominal pain; neutropenia, leucopenia, anaemia, thrombocytopenia with Theracurmin + gemcitabine | Phase I/II (multiple completed trials); insufficient evidence for PC standard-of-care; investigational adjunct |
| **Luteolin** | BCL-2/BAX, GSK-3β, NF-κB p65, Cytochrome C, Caspase-3, *KRAS*, STAT3/pSTAT3, DPD, MMP2/7/9, VEGF, FAK, PI3K/AKT, p70S6K | BCL-2/BAX mitochondrial apoptosis; GSK-3β/NF-κB; STAT3/pSTAT3; VEGF/angiogenesis; EMT (↓STAT3, ↓MMPs); JNK/TNF-α; PI3K/AKT/p70S6K | Displaces BAX from BCL-2 → mitochondrial apoptosis; luteolin + gemcitabine: ↓*KRAS* 46%, ↓pNF-κB 27%, ↑Caspase-3 417% in orthotopic models; inhibits PanIN-to-PC progression via ↓pSTAT3/DPD; suppresses migration/invasion | No clinical trials in PC. Trials in hepatocellular carcinoma (OS/PFS prolongation) and ovarian cancer (40% incidence reduction) suggest anticancer potential. NCT03288298 (tongue SCC) – outcomes not reported | Low bioavailability; limited oral absorption; rapid glucuronidation/sulfation; no validated PC-specific pharmacokinetic data | Favourable safety in preclinical studies; no PC-specific clinical safety data available | Preclinical only for PC; early-phase trials in other tumour types; significant gap in PC oncological translation |
| **Quercetin** | PI3K/AKT/mTOR, NF-κB, COX-2, 5-LOX, cFLIP, JNK, STAT3, MMP2/7, β-catenin, Nrf2/SLC7A11/GPX4, N-cadherin, Vimentin, miR-let-7c, miR-200b-3p | PI3K/AKT/mTOR; NF-κB/inflammatory axis; TRAIL/cFLIP/JNK apoptosis; STAT3; EMT (E-cadherin↑, N-cadherin↓, vimentin↓); Notch/Numbl (miRNA); Wnt/β-catenin; Nrf2/ferroptosis axis | Overcomes TRAIL resistance; outperforms gemcitabine in apoptosis induction in vitro; delays tumour growth alone or + gemcitabine (orthotopic models); targets CSC β-catenin; induces ferroptosis via Nrf2/SLC7A11/GPX4; inhibits EMT and metastasis | No trials dedicated to PC. Phase I (i.v.) demonstrated tyrosine kinase inhibition in vivo but subtherapeutic concentrations. NCT01912820 (prostate, n=60): 1000 mg/day modulates oxidative stress/gene expression; no PC-specific data | Poor aqueous solubility; low systemic bioavailability; rapid conjugation (glycosidation, glucuronidation); dense desmoplastic stroma of PC impedes tumour penetration | Well-tolerated in general; diarrhoea noted in combination with resveratrol; renal toxicity risk at very high i.v. doses | Preclinical/Phase I in other cancers; no PC-dedicated trial; critical gap in clinical development |
| **Resveratrol** | AKT/mTOR, BCL-2/MCL-1, BAX/BIM/BAD, Caspase-3, NF-κB/COX-2, STAT3, Notch1/2, WNT/β-catenin, Cyclin D1/A, P21/P27, VEGF, MMP-9, FOXO1/FOXO3a, SIRT1/AMPK, Nrf2 | AKT/mTOR; Sirt1/AMPK/Nrf2; Notch; STAT3; WNT/β-catenin; Hedgehog (↓Ihh/Ptch/Smo1); p38-MAPK; ERK/PI3K dephosphorylation; NF-κB/COX-2 | Inhibits proliferation in PDAC cell lines dose-dependently; synergistic with gemcitabine in vitro and orthotopic models; KITC analogue inhibits ribonucleotide reductase; regulates insulin secretion in β-cells; activates FOXO transcription factors in orthotopic PC | Phase I (n=40 healthy volunteers): safe, no adverse events; plasma 539 ng/mL but rapid hepatic elimination (t½ 2–5 h); metabolites (monoglucuronides, sulfates) 3–8× higher than control. No dedicated Phase II/III trial in PC | High absorption with high inter-individual variability; rapid hepatic first-pass metabolism; short t½ (2–5 h); active metabolites accumulate; chemopreventive threshold requires ≥5 µmol/L; low parent compound bioavailability | Well-tolerated orally; no serious adverse events at ≤5 g/day; diarrhoea in combination with quercetin | Phase I safety established; no Phase II/III in PC; promising adjunct candidate pending dedicated trials |
| **Vitamin C (Ascorbic acid)** | GLUT-1, PKM2, GAPDH, EGFR/EGF, MAPK/ERK, PI3K/AKT, H_2_O_2_ (extracellular), Catalase (tumour deficiency), TET/JHDM (epigenetic), cancer stem cells (mitochondrial OXPHOS) | Pro-oxidant (H_2_O_2_ generation) → caspase-3/7 apoptosis; Warburg effect disruption (↓GLUT-1/↓PKM2 in *KRAS*-mutant tumors); EGF/EGFR-MAPK/ERK inhibition; PI3K/AKT inhibition;; epigenetic reprogramming (TET/JHDM) | Selectively cytotoxic to *KRAS/BRAF*-mutant cells (PC >90% *KRAS*-mutant); decreases cell viability and increases apoptosis *in vitro*; potentiates gemcitabine (>50% tumour reduction); fasting-mimicking diet + Vit. C synergy in *KRAS*-mutant PC/CRC*; in vivo* tumour volume 138 mm³ vs. 472 mm³ controls | Phase I (PACMAN): i.v. Vit. C + gemcitabine → median OS 13±2 months vs. 5.65 months historical control. Phase I (NCT00954525): Vit. C + gemcitabine + erlotinib → 7/9 stable disease, 2/9 progressive. Heterogeneous evidence. | Water-soluble; heat- and oxygen-sensitive; oral route limited by intestinal absorption saturation; i.v. administration achieves pharmacological (pro-oxidant) concentrations; rapid renal excretion; *KRAS*-mutant tumour selectivity via GLUT-1 | Well-tolerated i.v. in clinical trials; contraindicated in G6PD deficiency (haemolysis risk); potential for kidney stones at very high oral doses; no increased toxicity added to gemcitabine/erlotinib | Phase I completed; Phase II warranted; promising chemosensitiser; not standard-of-care; requires larger RCTs with biomarker stratification |
| **Vitamin D (Calcitriol/analogues)** | VDR/RXR heterodimer, P21/P27 (↑), N-cadherin (↓), Vimentin (↓), Snail/Slug (↓), MMP-2/MMP-9 (↓), AMPK, AKT (↓), PSC-VDR (stromal target), SLC7A11 (ferroptosis-CRC) | VDR/VDRE genomic signalling → G1/S arrest (↑P21/P27); EMT reversal (↓Snail/Slug/Vimentin/N-cadherin); stromal remodelling via PSC-VDR inhibition → desmoplasia reduction; AMPK activation with AICAR combination; ferroptosis (in CRC via SLC7A11) | ↑P21/P27 → G1/S arrest; MART-10 analogue: anti-proliferative + anti-migratory (*KRAS*-independent); paricalcitol inhibits PSC activation → stromal unbundling; cholecalciferol + calcium ↓normal pancreatic cell proliferation; 1-α,25(OH)2D3 reduces PC in *KRAS* mutant models | Phase II (seocalcitol/EB1089, n=36): tolerated, no OS/TTF benefit. Phase II (calcitriol + docetaxel, n=25): 3/25 PR, 7/25 SD, modest TTP extension. Pilot (NCT03883919, paricalcitol + nanoliposomal irinotecan + 5-FU/LV, n=20): safe; weight-based arm ↑tumour vascularity. Multiple trials ongoing. | Dietary forms (D2/D3) require sequential hepatic (→calcidiol) and renal (→calcitriol) hydroxylation; calcitriol active but causes hypercalcemia; paricalcitol avoids hypercalcemia; fat-soluble → accumulation risk; individual variation in 25-OH hydroxylation | Calcitriol: hypercalcemia limits clinical use; seocalcitol and paricalcitol: acceptable safety profile without hypercalcemia; no weight loss with MART-10 analogue | Phase II completed; multiple Phase I/II ongoing; not standard-of-care; warrants biomarker-driven trials (VDR expression in PSCs/tumour) |
| **Nimbolide** | RNF114 (E3 ubiquitin ligase), PARP1, SOD2, PI3K, AKT, mTOR, ERK, BAX, Caspase-3, CD44, E-cadherin, N-cadherin, Snail, Notch-2, ROS | ROS-mediated mitochondrial apoptosis; PI3K/AKT/mTOR inhibition; ERK suppression; EMT inhibition; CSC targeting (CD44); oxidative stress via SOD2 inhibition; DNA damage response through RNF114; PARP1 trapping and synthetic lethality in *BRCA*-mutated cells | Potently inhibits PC cell proliferation *in vitro* and *in vivo*; induces mitochondrial ROS-dependent apoptosis and autophagy; suppresses EMT; reduces CD44⁺ cancer stem cells; PLGA-encapsulated nanoparticles enhance mesenchymal-to-epithelial transition through dual AKT/mTOR inhibition; potential synergy with PARP inhibitors and gemcitabine | No clinical trials in pancreatic cancer or any other malignancy to date. Translational rationale is particularly strong for *BRCA*1/2-mutated PDAC owing to RNF114 inhibition and PARP1 trapping but remains entirely preclinical. | Poorly characterized pharmacokinetics; low aqueous solubility expected for limonoid terpenoids; no long-term toxicology studies available; nanoformulations (PLGA nanoparticles) improve delivery and biological activity but remain experimental. | Generally, well tolerated in preclinical studies with no major toxicity reported at therapeutic doses; however, formal dose-escalation, chronic toxicity and safety pharmacology studies are lacking. | Preclinical only; no registered clinical trials. Requires pharmacokinetic optimization, toxicological evaluation and biomarker-driven studies, particularly in *BRCA*-mutant and *KRAS*-mutant PDAC. |

*Abbreviations: AKT, protein kinase B; AMPK, AMP-activated protein kinase; BAX/BCL-2, apoptosis regulators; CBD, cannabidiol; COX-2, cyclooxygenase-2; CSC, cancer stem cells; DPD, dihydropyrimidine dehydrogenase; EMT, epithelial-to-mesenchymal transition; ER, endoplasmic reticulum; FOXO, forkhead box O; G6PD, glucose-6-phosphate dehydrogenase; GAPDH, glyceraldehyde-3-phosphate dehydrogenase; GLUT-1, glucose transporter-1; GPX4, glutathione peroxidase 4; HIF-1α, hypoxia-inducible factor 1-alpha; i.v., intravenous; JNK, c-Jun N-terminal kinase; KRAS, Kirsten rat sarcoma viral proto-oncogene; MAPK, mitogen-activated protein kinase; MMP, matrix metalloproteinase; mTOR, mechanistic target of rapamycin; NF-κB, nuclear factor kappa-light-chain-enhancer of activated B cells; Nrf2, nuclear factor erythroid 2-related factor 2; OS, overall survival; OXPHOS, oxidative phosphorylation; PC, pancreatic cancer; PDAC, pancreatic ductal adenocarcinoma; PDH, pyruvate dehydrogenase; PD-L1, programmed death-ligand 1; PI3K, phosphoinositide 3-kinase; PKM2, pyruvate kinase isoform M2; PR, partial response; PSC, pancreatic stellate cell; QoL, quality of life; RCT, randomized controlled trial; ROS, reactive oxygen species; SD, stable disease; SIRT1, sirtuin 1; STAT3, signal transducer and activator of transcription 3; t½, half-life; TCA, tricarboxylic acid; TET, ten-eleven translocation; THC, tetrahydrocannabinol; TTP, time to progression; TTF, time to treatment failure; VDA, vitamin D analogue; VDR, vitamin D receptor; VEGF, vascular endothelial growth factor.*
